# Effects of plant protease inhibitors (Pep-3-EcTI, Pep-BbKI, and Pep-BrTI) versus corticosteroids on inflammation, remodeling, and oxidative stress in an asthma–COPD (ACO) model

**DOI:** 10.3389/fphar.2024.1282870

**Published:** 2024-05-07

**Authors:** Juliana Morelli Lopes Gonçalves João, Jéssica Anastácia Silva Barbosa, Luana Laura Sales da Silva, Silvia Fukuzaki, Elaine Cristina de Campos, Leandro do Nascimento Camargo, Tabata Maruyama dos Santos, Suellen Karoline Moreira Bezerra, Francine Maria de Almeida, Beatriz Mangueira Saraiva-Romanholo, Fernanda Degobbi Tenorio Quirino dos Santos Lopes, Camila Ramalho Bonturi, Renato Fraga Righetti, Maria Luiza Vilela Oliva, Iolanda de Fátima Lopes Calvo Tibério, Edna Aparecida Leick

**Affiliations:** ^1^ Faculdade de Medicina FMUSP, Universidade de São Paulo, São Paulo, Brazil; ^2^ University City of São Paulo, São Paulo, Brazil; ^3^ Departamento de Bioquímica, Universidade Federal de Sao Paulo (UNIFESP), São Paulo, Brazil; ^4^ Hospital Sírio-Libanês, São Paulo, Brazil

**Keywords:** asthma–chronic obstructive pulmonary disease overlap syndrome, serine proteinase Inhibitors, inflammation, oxidative stress, airway remodeling, dexamethasone

## Abstract

The peptide derived from *E. contortisiliquum* trypsin inhibitor (Pep-3-EcTI), peptide derived from kallikrein inhibitor isolated from *B. bauhinioides* (Pep-BbKI), and *B. rufa* peptide modified from *B. bauhinioides* (Pep-BrTI) peptides exhibit anti-inflammatory and antioxidant activities, suggesting their potential for treating asthma–chronic obstructive pulmonary disease (COPD) overlap (ACO). We compared the effects of these peptides with dexamethasone (DX) treatment in an ACO model. In this study, 11 groups of male BALB/c mice were pre-treated under different conditions, including sensitization with intraperitoneal injection and inhalation of ovalbumin (OVA), intratracheal instillation of porcine pancreatic elastase (ELA), sensitization with intraperitoneal injection, and various combinations of peptide treatments with Pep-3-EcTI, Pep-BbKI, Pep-BrTI, dexamethasone, and non-treated controls (SAL-saline). Respiratory system resistance, airway resistance, lung tissue resistance, exhaled nitric oxide, linear mean intercept, immune cell counts in the bronchoalveolar lavage fluid, cytokine expression, extracellular matrix remodeling, and oxidative stress in the airways and alveolar septa were evaluated on day 28. Results showed increased respiratory parameters, inflammatory markers, and tissue remodeling in the ACO group compared to controls. Treatment with the peptides or DX attenuated or reversed these responses, with the peptides showing effectiveness in controlling hyperresponsiveness, inflammation, remodeling, and oxidative stress markers. These peptides demonstrated an efficacy comparable to that of corticosteroids in the ACO model. However, this study highlights the need for further research to assess their safety, mechanisms of action, and potential translation to clinical studies before considering these peptides for human use.

## 1 Introduction

Asthma affects approximately 300 million people globally, resulting in significant public health and societal costs. Chronic obstructive pulmonary disease (COPD) is a major public health concern and a leading cause of chronic morbidity and mortality worldwide, and its impact is expected to increase owing to an aging population and persistent risk factors (Global Initiative for Asthma, 2022; Global Initiative for Chronic Obstructive Lung Disease, 2022).

In recent years, overlapping features have been observed in asthma and COPD, termed asthma–COPD overlap (ACO), with symptoms similar to those of COPD but with a significant response to bronchodilators, as in asthma. ACO may be present in 10%–40% of patients with COPD and 15%–35% of patients with asthma. Each condition may involve distinct inflammatory patterns and underlying mechanisms, although some commonalities may exist between them (Yanagisawa and Ichinose, 2018; Mekov et al., 2021).

Studies indicate that ACO exhibits a blend of inflammatory patterns, expressing Th2 cytokines such as IL-13 and IL-5. These cytokine levels are the highest in asthma, intermediate in ACO, and lowest in COPD (de Llano et al., 2018).

Smokers with asthma display increased airway neutrophilia, such as COPD, and elevated levels of cytokines, such as IL-6, IL-8, and IL-17. IL-17 plays a role in both neutrophilic bronchial asthma and the secretion of matrix metalloproteinase (MMP-9) by macrophages in COPD. Smokers with asthma also show increased bronchial infiltration of CD8+T cells, macrophages, and epithelial remodeling, similar to COPD (Siew et al., 2017; Ravensberg et al., 2013).

Individuals diagnosed with ACO are at a higher risk of exacerbations, which are often characterized by severe hypoxia resulting from irreversible airway obstruction and compromised alveolar diffusion capacity due to emphysematous changes. These factors significantly impact morbidity and mortality, leading to a notable decline in the quality of life for these patients (Yanagisawa and Ichinose, 2018; Global Initiative for Asthma, 2022; Global Initiative for Chronic Obstructive Lung Disease, 2022).

Protease inhibitors, abundant in nature, are found in animals, plants, and microorganisms, with plants containing them in significant amounts, typically constituting 1%–10% of total protein content in storage organs like seeds and tubers (Bonturi et al., 2022). One of their key roles is to inhibit proteolytic enzymes or increase endogenous anti-proteases to prevent disease progression. Proteases, acting as signaling molecules, participate in various processes such as hemostasis, cell death, cell proliferation, DNA replication, inflammatory responses, and tissue remodeling. They can regulate apoptosis and the cell cycle (Ferreira et al., 2013; Martins-Olivera et al., 2016).

Kunitz-type plant inhibitors belong to a significant family of protease inhibitors and typically weigh 20–22 kDa. They consist of one or two polypeptide chains linked by disulfide bonds and may lack or contain cysteine residues. These inhibitors serve as defense proteins with antibacterial, antifungal, anti-inflammatory, anticoagulant, antithrombotic, and anticancer activities. They vary in structure, with some being single-chain proteins and others being double-chain proteins with disulfide bridges. Examples include *Bauhinia bauhinioides* kallikrein inhibitor (BbKI), a single-chain inhibitor with a cysteine residue, and *Bauhinia rufa* trypsin inhibitor (BrTI), a single-chain inhibitor without cysteine residues derived from BbKI. *Enterolobium contortisiliquum* trypsin inhibitor (EcTI) is a double-chain inhibitor with four cysteines (Bonturi et al., 2022).

The first plant protease inhibitor isolated was *E. contortisiliquum*, known as EcTI. It belongs to the Leguminosae family and the Mimosoidea subfamily. Found in Brazil, it originates from a large tree and is popularly known by various names, such as Monkey’s Ear, Timbaúba, Ximbó, and Tamburé, among others (Zhou et al., 2013). Maria Luiza Vilela Oliva, from the Department of Biochemistry at the Federal University of São Paulo (UNIFESP), isolated the seeds of *E. contortisiliquum* and extracted the EcTI. EcTI has a molecular mass of 20,000 Da. Its function is to inhibit the activity of trypsin, chymotrypsin, human plasma kallikrein (HuPK), plasmin, and human neutrophil elastase and prevent the activation of MMP-2 and MMP-9 (Oliva and Sampaio, 2009; Nakahata et al., 2011). This inhibitor has a high affinity for trypsin, chymotrypsin, neutrophil elastase, HuPK, plasmin, and factor Xlla in the coagulation cascade. As a result of these properties, the inhibitor can inhibit blood clotting and the activity of fibrinolytic enzymes (Theodoro Junior et al., 2017).

Approximately 570 species in the *Bauhinia* genus are known; some are popularly referred to as “Cow’s Hoof” or “Ox Claw.” Pharmacological and phytochemical studies have demonstrated that their main constituents are glycosides, steroids, triterpenes, lactones, and flavonoids (Oliva and Sampaio, 2009; Almeida-Reis et al., 2017). The BbKI inhibitor is a potent blocker of serine proteases, especially HuPK, one important function of which is to release kinins involved in inflammation (Bonturi et al., 2022). Kallikreins are glycoproteins found in tissues, neutrophils, glandular cells, and biological fluids, with functions such as ion transport, glucose transport in synergy with insulin, and the regulation of blood flow through kinins (Martins-Olivera et al., 2016).

Prof. Maria Luiza Vilela Oliva’s group, from the Department of Biochemistry at UNIFESP, isolated from the seeds of *B. rufa*, the protease inhibitor *B. rufa* trypsin inhibitor (BrTI), whose structure is distinguished from other inhibitors by presenting the sequences of the arginine-glycine-aspartic acid (RGD) and arginine–glycine–glutamic acid (RGE) tripeptides, which are related to the inhibition of cell adhesion (Hostetter, 2000; Ruoslahti and Pierschbacher, 1986). BrTI is formed by a single, 20-kDa polypeptide chain without cysteine residues in its primary structure or an arginine residue in its reactive site. This inhibitor specifically inhibits human plasma trypsin and kallikrein (Nakahata et al., 2006). This research group has already studied the effects of synthetic peptides that make up the triad in several pathophysiological processes, such as triple-negative breast cancer (Valente, 2023) and thrombosis (Medina et al., 2022). In the current work, we studied the Pep-BrTI peptide that contains an RGE sequence.

According to Yavari et al. (2018), these peptides offer minimal immunogenicity, excellent tissue penetrability, low-cost manufacturing, and ease of modification to increase their *in vivo* stability and biological activity.

The control of asthma symptoms typically involves corticosteroid anti-inflammatories combined with β2-adrenergic agonists; however, some patients exhibit poor response to corticosteroid treatment, requiring higher doses associated with increased adverse effects. Additionally, the inflammatory response in COPD is often resistant to corticosteroids and shows a reduced sensitivity to bronchodilators. Despite the efficacy of current medications, a significant proportion of patients with asthma and COPD do not respond well or experience adverse effects (Johnson, 2004). Recent clinical strategies have targeted proteases as crucial signaling molecules in inflammation. Protease inhibitors such as EcTI and BbKI have demonstrated anti-inflammatory effects in experimental models; however, the actions of Pep-3-EcTI, Pep-BbKI, and Pep-BrTI remain unexplored. This study aimed to investigate the pathophysiological mechanisms of ACO and evaluate the potential therapeutic efficacy of Pep-3-EcTI, Pep-BbKI, and Pep-BrTI as treatment options for this condition.

## 2 Materials and methods

### 2.1 Animals

The study protocol was approved by the Ethics Committee of the University of São Paulo (process number: 1030/2018). Male BALB/c mice, aged 6–8 weeks and weighing 25–30 g, were obtained from the University of São Paulo, housed in an animal facility with a 12-h light-dark cycle, and provided with water and chow (National Research Council, 2011). The animals received humane care according to the “Guide for Care and Use of Laboratory Animals” (National Institutes of Health, publication 86–23, revised 1985). This study was conducted at the Laboratory of Experimental Therapy I (LIM-20) of the Faculty of Medicine, University of São Paulo, and was financially supported by FAPESP (grant number 2018/02537-5) and CNPq.

### 2.2 Peptide expression

The peptides were designed and synthesized based on the sequences of the EcTI, BbKI, and BrTI proteins, establishing the smallest structure responsible for inhibitory function and correlating with the structure and specificity of action of each protein. The Pep-3-EcTI peptide sequence is in the process of being patented and, therefore, will not be disclosed in this article. Peptide BbKI (Pep-BbKI) had the RPGLPVRFESPL sequence. Peptide BrTI (Pep-BrTI), in contrast, has its RGD (Ac-Y-L-E-P-V-A-R-G-D-G-G-L-A-NH2) and RGE (Ac-I-V-Y-Y-P-D-R-G-E-T-G-L-NH2) sequences derived from the primary sequence of BrTI. They were synthesized with a degree of purity equal to or greater than 98% and evaluated by reverse phase chromatography on a high-performance liquid chromatography (HPLC) system. The synthesis was carried out by WatsonBio, Houston, TX, United States.

### 2.3 Experimental groups and study design

The animals were assigned to 11 groups, each consisting of eight animals, based on the specific protocol they underwent: a) SAL control group (received a saline protocol); b) ovalbumin (OVA) group (sensitization with intraperitoneal injection + inhalations of OVA); c) ELA group (intratracheal instillation of porcine pancreatic elastase); d) ACO group (sensitization with intraperitoneal injection + inhalations of ovalbumin + intratracheal instillation of porcine pancreatic elastase); e) ACO-Pep-3-EcTI group (sensitization with intraperitoneal injection + inhalations of OVA solution, intratracheal instillations of elastase, and treatment with synthetic peptide derived from EcTI); f) ACO-Pep-BbKI group (sensitization with intraperitoneal injection + inhalations of OVA solution, intratracheal instillations of elastase, and treatment with synthetic peptide derived from BbKI); g) ACO-Pep-BrTI group (sensitization with intraperitoneal injection + inhalations of OVA solution, intratracheal instillations of elastase, and treatment with synthetic peptide derived from Pep-BrTI); h) ACO-DX group (sensitization with intraperitoneal injection + inhalations of OVA solution, intratracheal instillations of elastase, and treatment with dexamethasone); i) control saline SAL-Pep-3-EcTI (received saline protocol and treated with Pep-3-EcTI); j) control saline SAL-Pep-BbKI (received saline protocol and treated with Pep-BbKI); and k) control saline SAL-Pep-BrTI (received saline protocol and treated with Pep-BrTI). Eight animals were lost to the study. These protocols, which lasted for 28 days, were adapted from Toledo et al. (2013) and Ikeda et al. (2014).

### 2.4 Protocol of the experimental model

#### 2.4.1 Asthma mouse model (ovalbumin-induced) and emphysema mouse model (elastase-induced)

**FIGURE 1:**
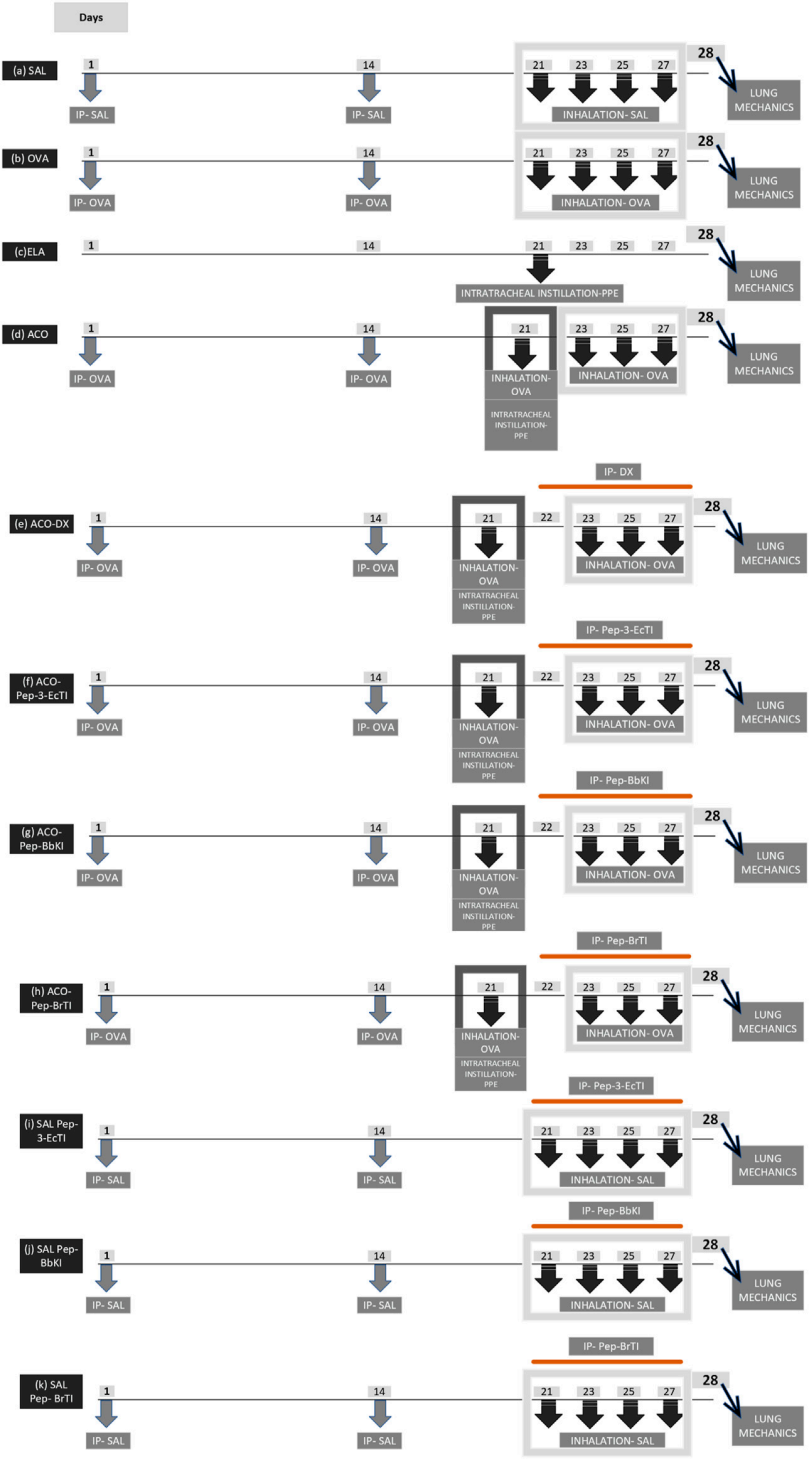
Illustrative scheme of the protocol of the experimental groups. I.P, intraperitoneal injection; I.T, intratracheal; PPE, porcine pancreatic elastase.

The protocol for sensitization and induction of pulmonary inflammation by ovalbumin (OVA group) lasted 28 days. The mice received a solution of 50 mg ovalbumin (A-5378, Sigma-Aldrich, St. Louis, MO, United States) emulsified in 6 mg aluminum hydroxide adjuvant (Pepsamar, Sanofi-Synthelabo SA, Rio de Janeiro, Brazil) intraperitoneal injection (I.P.) on days 1 and 14. Subsequently, on days 21, 23, 25, and 27, the animals were placed in an acrylic display box coupled with an ultrasonic nebulizer (US–1000; ICEL, São Paulo, Brazil) and subjected to aerosol inhalation of OVA diluted in NaCl 0.9% (saline) at a concentration of 10 mg/mL (1%) for 30 min. The control group (SAL) received a sterile saline solution instead of OVA (Figure 1). The doses were determined as described in a previous study by Toledo et al. (2013).

For the protocol of the ELA group, the mice were anesthetized with inhaled isoflurane 1 mL/mL (Isofurine^®^, Cristália LTDA, Itapira, SP, Brazil) and received intratracheal instillation of porcine pancreatic elastase (PPE) (E1250-500 mg elastase from porcine pancreas type I, ≥4 units/mg protein, 41.7 mL, 12 mg protein/mL; 5 units/mg protein; Sigma-Aldrich, St Louis, MO, United States) on day 21 of the experimental protocol—at a dose and concentration of 25 U PPE/100 g body weight dissolved in 40 μL of normal saline (Figure 1), as previously described by Ikeda et al. (2014). All animals were evaluated after 7 days of elastase treatment.

#### 2.4.2 Experimental model of ACO

The mice in this experimental group were subjected to two previously described experimental protocols (an asthma model and an elastase-induced lung injury) following the dose and date protocols illustrated in Figure 1 (Toledo et al., 2013; Ikeda et al., 2014; Souza et al., 2018).

Therefore, the mice received a solution of 50 mg OVA (Sigma-Aldrich) and 6 mg aluminum hydroxide (Alumen, Pepsamar, Sanofi-Synthelabo S.A., Rio de Janeiro, Brazil) I.P on days 1 and 14.

On days 21, 23, 25, and 27, the animals were placed in an acrylic exposure box coupled with an ultrasonic nebulizer (US-1000; ICEL, São Paulo, Brazil) and subjected to inhalation of OVA aerosols diluted in 0.9% NaCl (physiological serum) at a concentration of 10 mg/mL (1%). The duration of aerosol exposure was 30 min.

On day 21 of the protocol, the mice were anesthetized with inhaled isoflurane (Isofurine^®^) and instilled with PPE (EMD Chemicals) at a dose and concentration of 25 U PPE/100 g of body weight dissolved in 40 μL of saline, intratracheally. The mice were administered elastase and OVA. Elastase was administered 6 h after OVA inhalation.

#### 2.4.3 Treatment protocol with peptides (EcTI, BbKI, and BrTI) and dexamethasone

Pep-3-EcTI, Pep-BbKI, and Pep-BrTI were administered at a dose of 2 mg/kg I.P, 1 hour before each aerosol administration of OVA on days 22, 23, 25, and 27 of the ACO protocol experiment. On the same days as the protocol, the group treated with corticosteroids received intraperitoneal injections of 5 mg/kg DX (Aché Laboratórios Farmacêuticos S.A., São Paulo, Brazil), as described previously by Toledo et al. (2013). The treatments were administered 1 hour before each OVA nebulization when asthma was induced in the experimental group according to the protocol (Figure 1).

### 2.5 Lung function and exhaled nitric oxide collection

The methacholine dose–response curve and collection of exhaled nitric oxide (eNO) occurred 24 h after the completion of the experimental protocol on day 28. The mice were anesthetized with thiopental (50 mg/kg, I.P) and tracheostomized. The animal was connected to a mechanical ventilation device for small animals (flexiVent; Scireq, Montreal, Canada) and ventilated with a tidal volume of 10 mL/kg at a respiratory rate of 150 cycles/min with a sinusoidal inspiratory flow curve. To determine the exhaled nitric oxide (eNO), an impermeable balloon was connected to the expiratory port of the ventilator (Mylar Bag; Sievers Instruments Inc., Boulder, CO, United States). After collecting for 10 min, the balloon was sealed for further analysis. The mean eNO concentration was recorded in parts per billion (ppb) as an index of NO concentration in the exhaled air. Exhaled nitric oxide was measured by chemiluminescence using a rapid response analyzer (208 NOA Nitric Oxide Analyzer; Sievers Instruments Inc., Boulder, CO, United States).

Baseline measurements were taken after 30 s of mechanical ventilation. A challenge was then conducted with methacholine inhalation at doses of 3 mg/mL, 30 mg/mL, and 300 mg/mL in the first, second, and third minutes, respectively. A percentage increase in parameters was obtained compared to the baseline value of methacholine.

The parameters used to describe the lung model were the resistance of the respiratory system (Rrs), elastance of the respiratory system (Ers), resistance of the larger airways (Raw), resistance of the smaller airways or tissues (Gtis), and elastance of the lung tissues (Htis) (Toledo et al., 2013). At the end of the evaluation, the abdomen was opened, and intravenous blood was collected from the inferior vena cava using a heparinized syringe (1000 IU). Subsequently, the anterior chest wall was opened for in-block removal of the lungs and heart for morphometric and histological analyses.

### 2.6 Collection and analysis of bronchoalveolar lavage fluid

Immediately after the mechanics, bronchoalveolar lavage was performed by infusing 0.5 mL of saline (0.9% saline solution) three times consecutively (total volume of 1.5 mL) through the tracheal cannula with a syringe. The recovered volume was centrifuged at 1000 revolutions per minute RCF g) at 5°C for 10 min; the recovery average was 80%. The cell bud was resuspended in 200 μL of saline. The total cell count was determined by optical microscopy using a Neubauer hemocytometer (400×). For differential counting, 100 μL of BAL was cytocentrifuged at 790 g for 6 min, and, after drying, the slide was stained using the Diff-Quick technique. The differential cell count was determined by evaluating 300 cells/slide using an optical microscope with an immersion objective (1000×) (Martins-Olivera et al., 2016). At the end of the evaluation, the lungs were removed in a bloc with the heart for morphometric and histological/histochemical analyses.

### 2.7 Histology and immunohistochemistry

After fixation, the material was subjected to the usual histological techniques with paraffin to obtain 4-µm-thick sections, and the slides were stained as described below in Table 1.

**TABLE 1:** Markers, specifications, and dilutions. IL, interleukin; IFN, interferon; TNF, tumor necrosis factor; MMP, matrix metalloprotease; TGF, transforming growth factor; iNOS, inducible nitric oxide synthase; NF-kappaB, nuclear factor kappaB.

Marker	Primary antibody specification	Dilution	Secondary antibody	Second antibody specification
IL-1β	SC-52012, L: A0719; Sta Cruz Biotechnology, CA, United States	1:50	Anti-mouse	L: ZG0715, Vector; Vectastain Elite ABC Kit Peroxidase (Mouse IgG), CA, United States
IL-4	SC-53084, L: J1518; Sta Cruz Biotechnology, CA, United States	1:8000	Anti-mouse	L: ZF0206, Vector; Vectastain Elite ABC Kit Peroxidase (Mouse IgG), CA, United States
IL-5	SC-398334, L: F1617; Sta Cruz Biotechnology, CA, United States	1:300	Anti-mouse	L: ZF0206, Vector; Vectastain Elite ABC Kit Peroxidase (Mouse IgG), CA, United States
IL-6	LS-C746886, L: 144178; LSBio, WA, United States	1:200	Anti-rabbit	L: ZF0103, Vector; Vectastain Elite ABC Kit (Rabbit IgG), CA, United States
IL-10	SC-8438. Sta Cruz Biotechnology, CA, United States	1:50	Anti-mouse	L: ZF0206, Vector; Vectastain Elite ABC Kit Peroxidase (Mouse IgG), CA, United States
IL-13	SC-393365, L: G1715; Sta Cruz Biotechnology, CA, United States	1:8000	Anti-mouse	L: ZF0206, Vector; Vectastain Elite ABC Kit Peroxidase (Mouse IgG), CA, United States
IL-17	SC-7927, L: A3113; Sta Cruz Biotechnology, CA, United States	1:100	Anti-rabbit	L: ZF0103, Vector; Vectastain Elite ABC Kit (Rabbit IgG), CA, United States
IFN- γ	SC-8308; L: B2811; Sta Cruz Biotechnology, CA, United States	1:100	Anti-rabbit	L: ZF0103, Vector; Vectastain Elite ABC Kit (Rabbit IgG), CA, United States
TNF-α	SC-52746, L: J2418; Sta Cruz Biotechnology, CA, United States	1:5000	Anti-mouse	L: ZF0206, Vector; Vectastain Elite ABC Kit Peroxidase (Mouse IgG), CA, United States
MMP-9	SC-393859, L: 6118; Sta Cruz Biotechnology, CA, United States	1:800	Anti-mouse	L: ZF0206, Vector; Vectastain Elite ABC Kit Peroxidase (Mouse IgG), CA, United States
MMP-12	SC-30072; L: B1910; Sta Cruz Biotechnology, CA, United States	1:400	Anti-rabbit	L: ZF0103, Vector; Vectastain Elite ABC Kit (Rabbit IgG), CA, United States
TGF-β	SC-130348, L: A0219; Sta Cruz Biotechnology, CA, United States	1:700	Anti-mouse	L: ZF0206, Vector; Vectastain Elite ABC Kit Peroxidase (Mouse IgG), CA, United States
iNOS	RB-9242-P, L: 9242P709C; Thermo Fisher Scientific, United Kingdom	1: 150	Anti-rabbit	L: ZF0103, Vector; Vectastain Elite ABC Kit (Rabbit IgG), CA, United States
SOD3	SC-32222, L: E311; Sta Cruz Biotechnology, CA, United States	1:100	Anti-goat	L:41573742, Agilent; EnVision FLEX/HRP Kit, Glostrup, DK
NF-KappaB	SC-8008, L: B1119; Sta Cruz Biotechnology, CA, United States	1:700	Anti-mouse	L: ZF0206, Vector; Vectastain Elite ABC Kit Peroxidase (Mouse IgG), CA, United States

For picrosirius staining (collagen fibers), the sections were dewaxed and washed in water. Then, the sections were stained for 1 h with picrosirius red at room temperature and washed under running water for 5 min. Next, the sections were stained with Harris hematoxylin for 6 min and washed under running water for 10 min.

The immunohistochemical labeling procedures of the other samples were performed in the following sequence: antigen retrieval, blocking, incubation with the primary antibody, incubation with the secondary antibody complex, staining, and contrast. The process is described as follows: first, deparaffinization, followed by hydration, digestion or antigen retrieval in the pressure cooker for 1 min at 125°C (using citrate pH 6 or EDTA pH 9 buffers). After this step, peroxidase blocking was performed using 10 volumes of hydrogen peroxide (3%) for 5 min and washing three times with PBS. The diluted antibodies were pipetted onto the tissue, and the slides were incubated in a humid chamber overnight (18–20 h).

After the slides were incubated in the refrigerator and washed with PBS, they were incubated in a humid chamber with the secondary antibody vector (rabbit, rat, goat, or mouse) for 30 min at 37°C and then washed with PBS three times for 3 min each time. Subsequently, the slides were incubated in a humid chamber with the Vector AB Conjugate Complex for 30 min at 37°C, washed again in PBS, and developed in the chromogenic solution (70 mg of DAB per 110 mL of tris-HCl). The sections were stained with Harris hematoxylin, and the slides were mounted. Immunohistochemistry was performed for several markers. Cytokine and NF-kappaB are found in inflammatory cells, including interleukin (IL)-1β, IL-4, IL-5, IL-6, IL-10, IL-13, IL-17, MMP-9, MMP-12, TNF-α, IFN-γ, NF-kappaB, TGF-β, SOD3, and inducible nitric oxide synthase (iNOS).

After these procedures, lung sections were analyzed using the morphometric technique described below.

### 2.8 Morphometric analysis

For the analysis of collagen fibers stained with picrosirius red, images were captured at a magnification of 400× using a microscope (Leica DM2500; Leica Microsystems, Wetzlar, Germany) coupled to a digital camera (Leica DFC420; Leica Microsystems, Wetzlar, Germany) using Infinity Capture software (Lumenera Corporation, Canada). We measured collagen fibers in the total area of the alveolar septa and around the airways, which consisted of 10–12 microscopic fields per lung. Images were captured and processed using the Qwim Plus program (Leica), and analyses were performed using Image ProPlus 4.5 software (NIH, MD, United States), which allowed the development of a color tone spectrum threshold. The shaded areas represent the positively quantified areas within the predetermined area. Optical cell density was determined and expressed as a percentage based on the relationship between the quantity of collagen fibers in a specific frame and the total area of that frame (volume fraction) (Righetti et al., 2014; Camargo et al., 2018; Camargo et al., 2023).

Eosinophils and inflammatory markers were counted by morphometry, where the point counting technique was used with a reticle of a known area (10^4^ μm^2^ at a magnification of 1000×) containing 50 lines and 100 points, coupled to an eyepiece at the ocular of a microscope (E200Mv, Nikon Corporation, Tokyo, Japan). Airway analysis was performed in four fields around three airways per animal. For the alveolar septa, analysis of 10 random lung fields was performed. All analyses were conducted at a magnification of 1000×. The number of points covering the lung tissue and the number of inflammation-positive cells were counted, resulting in the proportion of positive cells per tissue area. The result was expressed as positive cells/10^4^µm^2^.

### 2.9 Evaluation of the mean linear intercept

Point counting was used to quantify the mean linear intercept (Lm) (Theodoro-Júnior et al., 2017). The Lm determines the index of the mean diameter of the distal air spaces, indicating the degree of alveolar distension (Margraf et al., 1991; Hsia et al., 2010). The reticulum was attached to an optical microscope (E200Mv, Nikon Corporation, Tokyo, Japan), and the analysis was performed by counting the intersections between the reticulum lines and the lung parenchyma. Twenty distinct random fields of the lung parenchyma were counted at 200× magnification on slides stained with hematoxylin and eosin. Lm was calculated using the equation: Lm = 2,500 μm/number of times the line and the alveolar septum intersect.

### 2.10 Statistical analysis

Parametric data are presented as mean ± standard error and graphs in bar format. *One-way analysis of variance* (ANOVA) was used, followed by the *Holm–Sidak method*, which was used to determine statistically significant differences between groups. We used SigmaPlot 11.0 software (Systat Software, SPSS Inc., United States) for all analyses, and statistical significance was set at *p* < 0.05.

## 3 Results

The SAL and SAL-peptide control groups were not significantly different (*p* > 0.05), as shown in Table 2. Therefore, only the SAL group is shown in the graphs to facilitate visualization of the results.

**TABLE 2:** Comparison of results of hyperresponsiveness to methacholine in the bronchoalveolar lavage fluid cells, inflammatory markers, remodeling markers, oxidative stress markers, and signaling pathways in the airways and alveolar septa between the SAL, SAL-Pep-3-EcTI, SAL-Pep-BbKI, and SAL-Pep-BrTI control groups.

	SAL	SAL-Pep-3-EcTI	SAL-Pep-BbKI	SAL-Pep-BrTI	p*
**Hyperresponsiveness to methacholine (%)**					
Rrs	73.5 ± 8.9	171.9 ± 48.0	161.0 ± 14.4	62.3 ± 26.2	*p* = 0.060
Ers	80.0 ± 7.1	44.0 ± 19.7	45.2 ± 3.3	88.5 ± 33.5	*p* = 0.147
Gtis	52.1 ± 9.3	45.8 ± 6.6	85.2 ± 13.6	108.7 ± 52.8	*p* = 0.089
Htis	45.5 ± 1.2	16.5 ± 2.9	24.9 ± 3.4	49.4 ± 8.7	*P* ≤ 0.001
Raw	158.8 ± 36.4	201.2 ± 34.1	159.6 ± 35.9	157.1 ± 96.6	*p* = 0.861
eNO	13.3 ± 1.7	18.4 ± 2.5	18,9 ± 0.4	19,3 ± 3.7	*p* = 0.173
**Bronchoalveolar lavage fluid (x10** ^**4**^ **cells/mL)**					
Total cells	0.5 ± 0.04	0.5 ± 0.07	0.5 ± 0.07	0.7 ± 0.01	*p* = 0.246
Eosinophils	0.2 ± 0.04	0.1 ± 0.02	0.1 ± 0.03	0.1 ± 0.02	*p* = 0.443
Neutrophils	0.2 ± 0.03	0.1 ± 0.02	0.1 ± 0.03	0.1 ± 0.02	*p* = 0.691
Lymphocytes	0.2 ± 0.03	0.2 ± 0.03	0.1 ± 0.02	0.1 ± 0.02	*p* = 0.119
Macrophages	0.2 ± 0.04	0.2 ± 0.03	0.1 ± 0.03	0.1 ± 0.02	*p* = 0.308
**Inflammatory marker (cells/10** ^**4**^ **µm** ^**2**^ **)**					
IL-1-β in the airway	0.4 ± 0.1	0.7 ± 0.1	0.6 ± 0.1	0.9 ± 0.2	*p* = 0.179
IL-1-β in the alveolar septa	0.4 ± 0.2	0.8 ± 0.2	0.4 ± 0.2	0.6 ± 0.2	*p* = 0.274
IL-5 in the airway	1.5 ± 0.2	0.6 ± 0.2	1.2 ± 0.3	1.3 ± 0.2	*p* = 0.141
IL-5 in the alveolar septa	1.2 ± 0.2	0.9 ± 0.1	0.7 ± 0.2	0.9 ± 0.2	*p* = 0.171
IL-6 in the airway	0.7 ± 0.2	0.9 ± 0.2	0.7 ± 0.1	0.4 ± 0.1	*p* = 0.156
IL-6 in the alveolar septa	0.5 ± 0.2	0.6 ± 0.1	0.4 ± 0.1	0.1 ± 0.1	*p* = 0.063
IL-10 in the airway	2.1 ± 0.3	2.5 ± 0.7	2.0 ± 0.2	2.4 ± 0.6	*p* = 0.845
IL-13 in the airway	2.8 ± 0.3	3.8 ± 0.4	2.5 ± 0.3	2.5 ± 0.3	*p* = 0.069
IL-13 in the alveolar septa	2.8 ± 0.4	2.8 ± 0.4	2.0 ± 0.4	2.0 ± 0.3	*p* = 0.208
IL-17 in the airway	2.2 ± 0.2	1.4 ± 0.3	1.7 ± 0.2	1.4 ± 0.3	*p* = 0.093
IL-17 in the alveolar septa	1.7 ± 0.2	1.0 ± 0.2	0.9 ± 0.2	1.1 ± 0.3	*p* = 0.168
TNF-α in the airway	1.8 ± 0.4	0.8 ± 0.2	2.0 ± 0.3	1.9 ± 0.5	*p* = 0.059
TNF-α in the alveolar septa	1.7 ± 0.3	1.3 ± 0.3	2.0 ± 0.4	2.5 ± 0.4	*p* = 0.107
INF-γ in the airway	0.9 ± 0.2	0.2 ± 0.1	0.4 ± 0.1	0.7 ± 0.2	*p* = 0.059
INF-γ in the alveolar septa	0.7 ± 0.2	0.1 ± 0.1	0.5 ± 0.1	0.5 ± 0.2	*p* = 0.080
**Remodeling markers (cells/10** ^**4**^ **µm** ^**2**^ **)**					
MMP-9 in the airway	0.3 ± 0.04	0.8 ± 0.2	0.4 ± 0.1	0.5 ± 0.1	*p* = 0.084
MMP-9 in the alveolar septa	0.9 ± 0.1	1.0 ± 0.2	0.3 ± 0.1	0.9 ± 0.2	*p* = 0.057
MMP-12 in the airway	1.8 ± 0.3	1.1 ± 0.2	1.1 ± 0.2	1.4 ± 0.2	*p* = 0.226
MMP-12 in the alveolar septa	0.6 ± 0.2	1.1 ± 0.2	0.5 ± 0.1	0.9 ± 0.2	*p* = 0.112
**Oxidative stress markers(cells/10** ^**4**^ **µm** ^**2**^ **)**					
iNOS in the airway	3.3 ± 0.6	2.3 ± 0.3	2.2 ± 0.3	2.1 ± 0.2	*p* = 0.206
iNOS in the alveolar septa	2.2 ± 0.3	1.5 ± 0.2	2.1 ± 0.2	1.5 ± 0.3	*p* = 0.138
**Signaling pathway (cells/10** ^**4**^ **µm** ^**2**^ **)**					
NF-KappaB in the airway	0.5 ± 0.2	1.0 ± 0.2	1.0 ± 0.2	1.3 ± 0.2	*p* = 0.056
NF-KappaB in the alveolar septa	0.5 ± 0.2	0.9 ± 0.2	0.5 ± 0.1	1.1 ± 0.2	*p* = 0.055

Rrs, respiratory system resistance; Ers, respiratory system elastance; Raw, resistance of the larger airways; Htis, tissue elastance; Gtis, tissue resistance; IL, interleukin; IFN, interferon; TNF, tumor necrosis factor; MMP, matrix metalloproteinase; TGF, transforming growth factor; iNOS, inducible nitric oxide synthase; NF, nuclear factor. *p-value.

### 3.1 Effects of treatments (dexamethasone and inhibitors of proteinase) on methacholine dose–response curve

**FIGURE 2:**
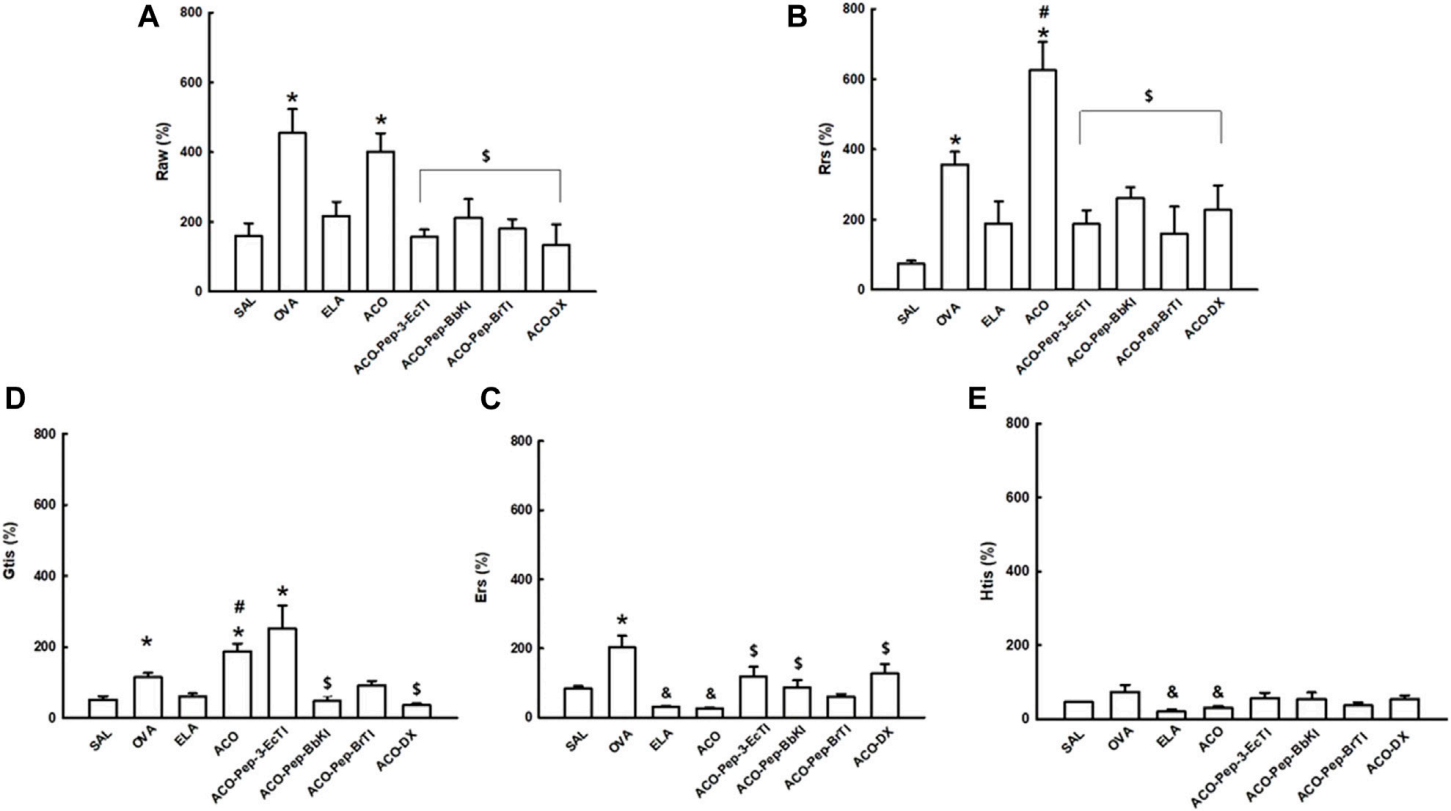
Mechanical evaluation of **(A)** airway resistance, **(B)** respiratory system resistance, **(C)** respiratory system elastance, **(D)** tissue resistance, and **(E)** lung tissue elastance. The results are expressed in percentages (%). *p < 0.05 compared to the SAL group; ^#^ *p* < 0.05 compared to the OVA and ELA groups; ^$^ *p* < 0.05 compared to the ACO group; ^&^ *p* < 0.05 compared to the OVA group.

Figure 2 and Table 3 show the results of bronchial hyperresponsiveness to methacholine. For %Rrs, %Raw, and %Gtis, the ACO and OVA groups showed increased values compared to the SAL group. In the %Gtis and %Rrs analyses, the ACO group showed increased values compared to the OVA and ELA groups (*p* < 0.05 for all comparisons). In the %Ers and %Htis analyses, the ACO and ELA groups were not different from the SAL group but presented lower values than the OVA group (*p* < 0.05 for all comparisons). When we analyzed the treatments with DX and the peptides (ACO-Pep-3-EcTI, ACO-Pep-BbKI, ACO-Pep-BrTI, and ACO-DX), we observed a total reversal when compared to the ACO group in %Raw, %Rrs, and % Gtis (*p* < 0.05 for all comparisons). Thus, it differs from the %Ers analysis, where the groups treated only with ACO-EcTI, ACO-BbKI, and ACO-DX showed a total reverse trend compared to the ACO group (*p* > 0.05 for all comparisons). The %Htis analysis of the treatment groups did not show a significant increase compared to the ACO group (*p* < 0.05 for all comparisons). The treated groups in all analyses did not differ among themselves or when compared with the SAL group (*p* < 0.05 for all comparisons).

**TABLE 3:** Raw, airway resistance, Rrs, respiratory system resistance, Ers, respiratory system elastance, Htis, lung tissue elastance, Gtis, and tissue resistance. The results are expressed in percentages (%). *p < 0.05 compared to the SAL group; ^#^ *p* < 0.05 compared to the OVA and ELA groups; ^$^ *p* < 0.05 compared to the ACO group; ^&^ *p* < 0.05 compared to the OVA group.

Bronchial hyperresponsiveness to methacholine								
Bronchial hyperresponsiveness to methacholine **(%)**	**SAL**	**OVA**	**ELA**	**ACO**	**ACO-Pep-3-EcTI**	**ACO-Pep-BbKI**	**ACO-Pep-BrTI**	**ACO-DX**
**Rrs**	73.5 ± 8.9	356.9 ± 36.5*	188.9 ± 63.4	625.1 ± 81.0*^/#^	186.7 ± 39.5^$^	260.7 ± 31.5^$^	159.5 ± 76.9^$^	227.3 ± 70.5^$^
**Ers**	90.6 ± 7.5	203.5 ± 33.8*	32.5 ± 3.2*^/&^	36.4 ± 4.9*^/&^	118.9 ± 28.1^$^	110.3 ± 28.8^$^	125.0 ± 64.6	112.5 ± 19.8^$^
**Raw**	158.8 ± 36.4	454.8 ± 68.6*	217.2 ± 40.4	402.1 ± 52.2*	157.0 ± 20.0^$^	238.0 ± 52.9^$^	224.5 ± 50.1^$^	133.0 ± 59.3^$^
**Htis**	45.5 ± 1.2	72.9 ± 19.0*	19.6 ± 5.4 ^&^	29.9 ± 5.7 ^&^	56.3 ± 14.8	53.8 ± 17.9	36.6 ± 8.1	54.1 ± 9.5
**Gtis**	35.4 ± 6.5	136.1 ± 24.2*	55.1 ± 8.4	176.4 ± 21.8*^/#^	93.1 ± 24.8*	47.8 ± 15.4^$^	87.6 ± 11.7	56.2 ± 17.2^$^

### 3.2 Bronchoalveolar lavage fluid

**FIGURE 3:**
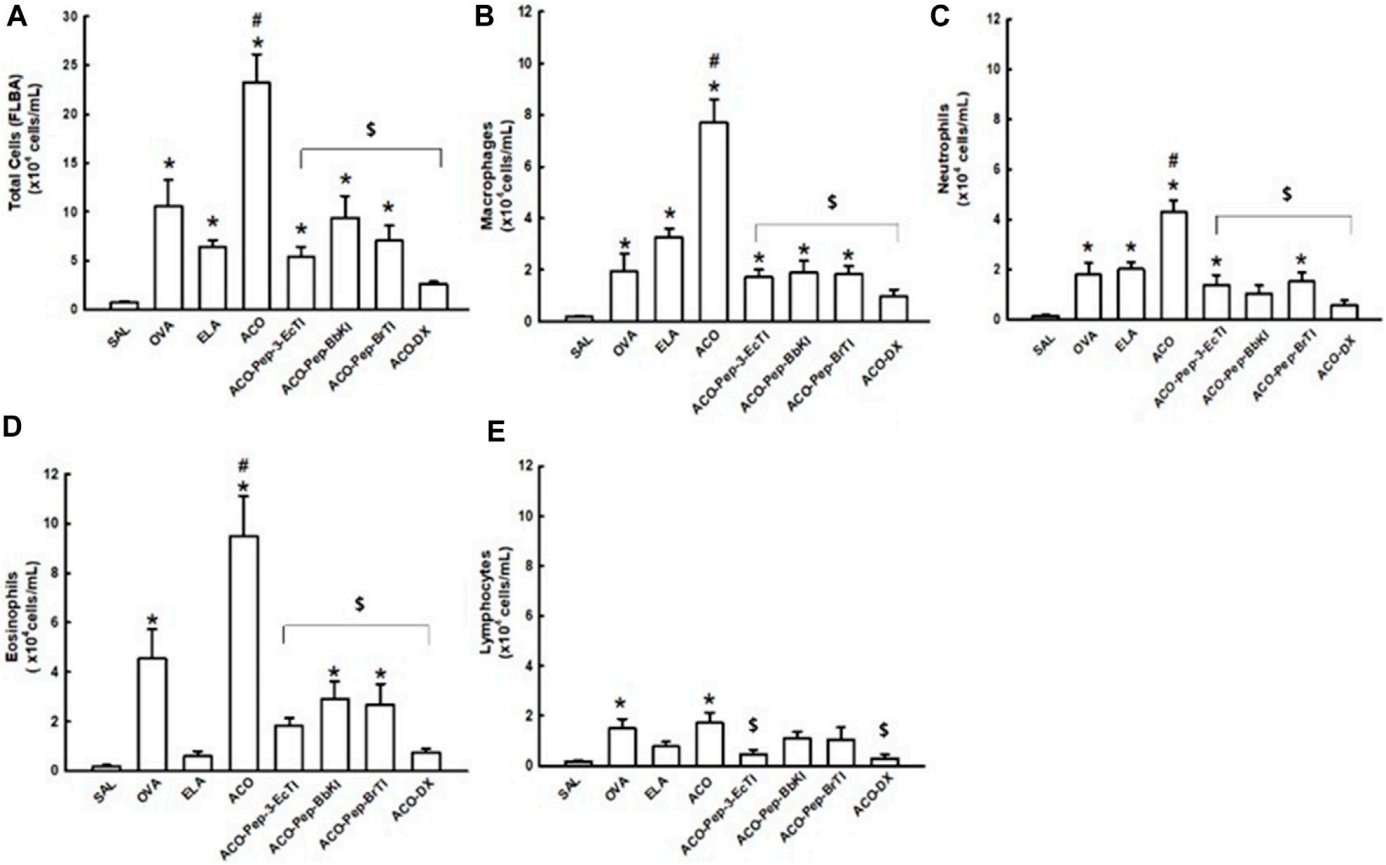
Evaluation of the number of cells in bronchoalveolar lavage fluid (BALF). The **(A)** total cells, **(B)** macrophages, **(C)** neutrophils, **(D)** lymphocytes, and **(E)** eosinophils are expressed in 10^4^ cells/mL. *p < 0.05 compared to the SAL group; ^$^ *p* < 0.05 compared to the ACO group; ^#^ *p* < 0.05 compared to the OVA and ELA groups.

Figure 3 shows the results of bronchoalveolar lavage, which showed an increase in total cells, as well as neutrophils, macrophages, eosinophils, and lymphocytes, in the ACO groups compared to the SAL group (*p* < 0.05 for all comparisons). In the analysis of the amounts of total cells, macrophages, eosinophils, and neutrophils, ACO showed an increased value when compared to the OVA and ELA groups (*p* < 0.05 for all comparisons). As for eosinophils, only the ACO and OVA groups showed increased values compared to the SAL group (*p* < 0.05 for all comparisons). The DX treatment groups showed total reversal, which was different from the ACO group (*p* < 0.05) and similar to the SAL group (*p* > 0.05), in the analyses of the amounts of total cells, macrophages, neutrophils, eosinophils, and lymphocytes. The groups treated with the peptides (ACO-Pep-3-EcTI, ACO-Pep-BbKI, and ACO-Pep-BrTI) showed attenuation, being different from ACO and SAL in the amounts of total cells and macrophages (*p* < 0.05 for all comparisons). In the analysis of neutrophils, the ACO-Pep-BbKI group showed attenuation in the amounts of total cells, macrophages, neutrophils, and eosinophils. In the neutrophil analysis, ACO-Pep-3-EcTI and ACO-Pep-BbKI peptides attenuated the response, in contrast to ACO and SAL (*p* < 0.05), and total reversal was observed with the ACO-BbKI peptide. In the eosinophil analysis, the ACO-Pep-BbKI and ACO-Pep-BrTI groups showed an attenuated response, differing from ACO and SAL, whereas the ACO-Pep-3-EcTI group was completely reversed, differing from ACO (*p* < 0.05) and similar to SAL (*p* > 0.05). The treatments in all analyses were not significantly different (*p* < 0.05 for all comparisons).

### 3.3 Qualitative analysis of the mean linear intercept (Lm)

**FIGURE 4:**
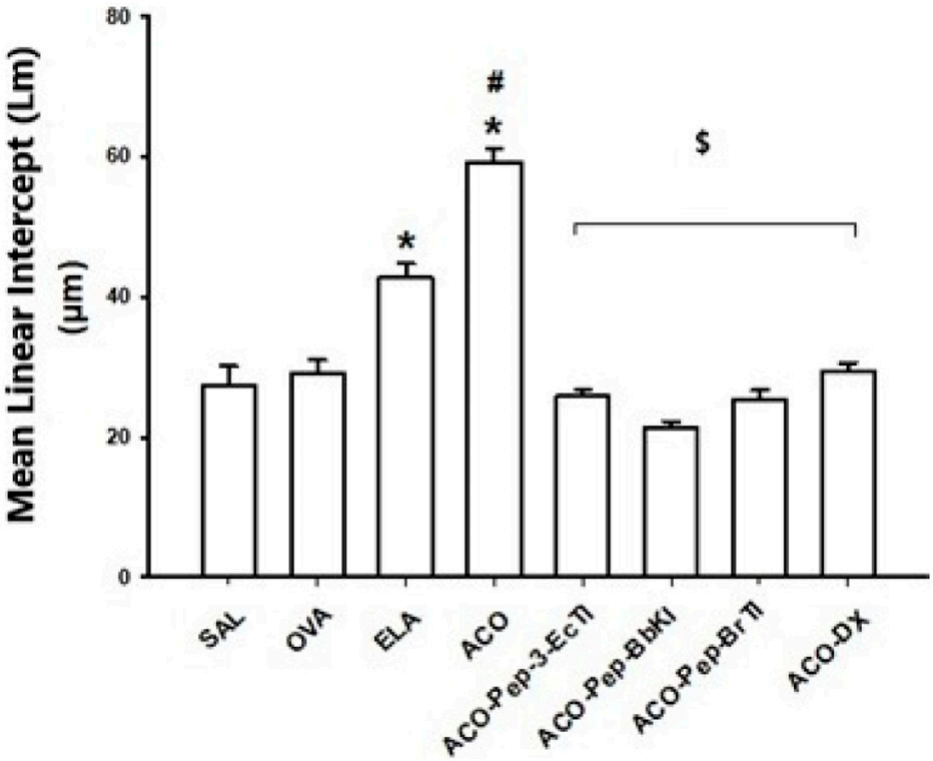
Effects of dexamethasone and peptide treatments in the qualitative analysis of the mean linear intercept (Lm); *p < 0.05 compared to the SAL group; ^$^ *p* < 0.05 compared to the ACO group; #*p* < 0.05, ACO compared to the OVA and ELA groups.

Figure 4 shows the evaluation of the Lm in the control groups SAL, OVA, ELA, and ACO and in the treatment groups ACO-Pep-3-EcTI (25.8% ± 0.9%), ACO-Pep-BbKI (21.4% ± 0.8%), ACO-Pep-BrTI (25.3% ± 1.3%), and ACO-DX (29.3% ± 1.3%). We observed an increase in Lm in the ELA (42.8% ± 1.9%) and ACO (59.2% ± 1.9%) groups when compared to the SAL group (27.2% ± 2.8%) (*p* < 0.05 for all comparisons). The ACO group is different from the OVA (29.2% ± 1.8%) and ELA (*p* < 0.05) groups. The results of the OVA group did not differ significantly from those of the SAL group. The change in Lm in the treated groups was reversed because they were different from the ACO group (*p* < 0.05 for both comparisons) and similar to that in the SAL group (*p* > 0.05 for both comparisons).

### 3.4 Lung inflammatory markers

**FIGURE 5:**
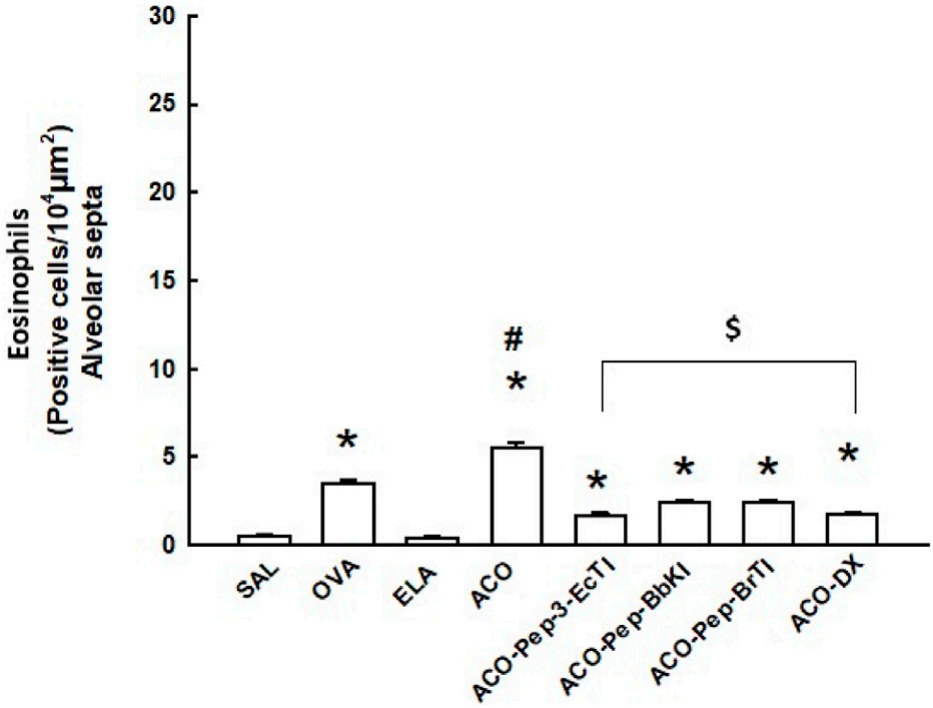
Effects of dexamethasone and peptide treatments on eosinophils (alveolar septa) in the SAL, OVA, ELA, ACO, ACO-Pep-3pEcTI, ACO-Pep-BbKI, ACO-Pep-BrTI, and ACO-DX groups; *p < 0.05 compared to the SAL group; $ *p* < 0.05 compared to the ACO group; #*p* < 0.05, ACO group compared to the OVA and ELA groups.

The results of inflammatory markers in the experimental groups are presented in Table 4 and Figure 5 for both the airways and alveolar septa. In the inflammatory marker results, a positive cell count was observed for IL-1β (AW/AS), IL-4 (AW/AS), IL-5 (AW/AS), IL-6 (AW/AS), IL-10 (AW/AS), IL-13 (AW/AS), IL-17 (AW/AS), INF-γ (AW/AS), and TNF-α (AW/AS), and ACO showed an increased response compared to the SAL group (*p* < 0.05). ACO was different from OVA and ELA in the analyses of IL-1β (AW/AS), IL-5 (AW/AS), IL-6 (AW/AS), IL-10 (AW), IL-13 (AS), INF-α (AW), and TNF-α (AS) (*p* < 0.05 for all comparisons). Analyzing the treatment groups, we observed that ACO-Pep-3-EcTI, ACO-Pep-BbKI, and ACO-Pep-BrTI attenuated the changes in the model, differing from ACO and SAL in IL-1β (AS), IL-6 (AS), and IL-13 (AW). In the following analyses, the ACO-Pep-3-EcTI, ACO-Pep-BbKI, and ACO-Pep-BrTI peptides completely reversed the alteration, being different from ACO (*p* < 0.05) and similar to SAL (*p* > 0.05) in TNF-α (AS), INF-γ (AW/AS), IL-17 (AW), IL-13 (AS), IL-10 (AW/AS), IL-6 (AW), IL-5 (AW)/AS), and IL-1 β(AS). The ACO-DX treatment group reversed changes from ACO to IL-1β (AW/AS), IL-4 (AS), IL-5 (AW/AS), IL-6 (AW/AS), IL-10 (AW/AS), IL-13 (AW/AS), IFN-γ (AW/AS), and TNF-α (AW/AS).

**TABLE 4:** IL, interleukin; TNF, tumor necrosis factor; IFN-γ, interferon-gamma. The results are expressed in positive cells/10^4^µm^2^ *p < 0.05 compared to the SAL group; ^$^ *p* < 0.05 compared to the ACO group; ^#^ *p* < 0.05, ACO group compared to the OVA and ELA groups.

Inflammatory markers								
Inflammatory marker (cells/10⁴µm^2^)	SAL	OVA	ELA	ACO	ACO-Pep-3-EcTI	ACO-Pep-BbKI	ACO-Pep-BrTI	ACO-DX
**IL-1β** in the **airway**	0.4 ± 0.1	4.2 ± 0.4*	1.2 ± 0.2	6.1 ± 0.6*^/#^	2.8 ± 0.4^$^	2.7 ± 0.3^$^	2.9 ± 0.3^$^	1.4 ± 0.3^$^
**IL-1β** in the **alveolar septa**	0.4 ± 0.2	2.6 ± 0.3*	0.8 ± 0.2	4.1 ± 0.3*^/#^	1.0 ± 0.2^$^	1.0 ± 0.3^$^	3.4 ± 0.5^$^	0.6 ± 0.2^$^
**IL-4** in the **airway**	1.6 ± 0.6	11.6 ± 1.3*	3.1 ± 0.9*	12.0 ± 2.0*	6.8 ± 0.9^$/^*	11.9 ± 1.3*	9.2 ± 1.1*	7.5 ± 0.9^$/^*
**IL-4** in the **alveolar septa**	1.8 ± 0.3	10.5 ± 0.6*	4.8 ± 0.7*	9.9 ± 0.7*	4.4 ± 0.5^$^	9.6 ± 0.6*	9.8 ± 0.7*	5.8 ± 0.4^$^
**IL-5** in the **airway**	1.5 ± 0.2	11.4 ± 0.8*	6.5 ± 0.5*	8.6 ± 0.7*^/#^	3.8 ± 0.4^$/^*	2.4 ± 0.4^$^	1.8 ± 0.2^$^	5.7 ± 0.4^$/^*
**IL-5** in the **alveolar septa**	1.2 ± 0.2	4.4 ± 0.4*	2.9 ± 0.6*	8.0 ± 0.5*^/#^	1.5 ± 0.3^$^	2.0 ± 0.3^$^	1.3 ± 0.3^$^	2.0 ± 0.2^$^
**IL-6** in the **airway**	0.7 ± 0.2	6.6 ± 0.4*	7.2 ± 0.5*	12.3 ± 1.1*^/#^	1.8 ± 0.3^$^	2.0 ± 0.3^$/^*	1.8 ± 0.3^$^	1.1 ± 0.2^$^
**IL-6** in the **alveolar septa**	0.5 ± 0.2	6.6 ± 0.4*	7.2 ± 0.5*	12.3 ± 1.1*^/#^	1.9 ± 0.3^$/^*	2.0 ± 0.3^$/^*	1.8 ± 0.3^$/^*	1.1 ± 0.2^$^
**IL-10** in the **airway**	2.1 ± 0.2	3.4 ± 0.3*	4.2 ± 0.4*	5.6 ± 0.4*^/#^	2.7 ± 0.4^$^	2.8 ± 0.3^$^	2.4 ± 0.3^$^	4.2 ± 0.4^$/^*
**IL-10** in the **alveolar septa**	3.3 ± 0.3	4.5 ± 0.3*	5.6 ± 0.4*	6.6 ± 0.5*	2.6 ± 0.5^$^	2.3 ± 0.2^$^	4.0 ± 0.5^$^	3.6 ± 0.4^$^
**IL-13** in the **airway**	2.9 ± 0.3	11.1 ± 0.8*	7.9 ± 0.7*	11.6 ± 0.6*	8.1 ± 0.6^$/*^	7.0 ± 0.7^$/^*	7.2 ± 0.6^$/^*	7.4 ± 0.6^$/^*
**IL-13** in the **alveolar septa**	3.2 ± 0.4	9.1 ± 0.6*	6.7 ± 0.6*	17.9 ± 1.8*^/#^	3.7 ± 0.3^$^	4.5 ± 0.4^$^	3.7 ± 0.4^$^	4.2 ± 0.4^$^
**IL-17** in the **airway**	2.2 ± 0.2	5.6 ± 0.3*	6.5 ± 0.3*	7.3 ± 0.5*	2.9 ± 0.4^$^	3.1 ± 0.3^$^	2.8 ± 0.3^$^	4.1 ± 0.3^$/^*
**IL-17** in the **alveolar septa**	1.7 ± 0.2	6.5 ± 0.3*	8.1 ± 0.5*	8.4 ± 0.6*	4.6 ± 0.7^$/*^	2.7 ± 0.2^$^	3.1 ± 0.3^$/^*	4.9 ± 0.3^$/^*
**TNF-α** in the **airway**	1.8 ± 0.4	6.2 ± 0.7*	5.3 ± 0.7*	4.9 ± 0.7*	4.2 ± 0.6	2.9 ± 0.4	4.8 ± 0.6	4.2 ± 0.7
**TNF-α** in the **alveolar septa**	1.7 ± 0.3	5.6 ± 0.5 *	6.2 ± 0.6*	9.2 ± 0.4*^/#^	2.3 ± 0.2^$^	2.2 ± 0.3^$^	2.6 ± 0.3^$^	3.5 ± 0.3^$/^*
**IFN-γ** in the **airway**	0.9 ± 0.2	2.1 ± 0.3	3.4 ± 0.4 *	6.3 ± 0.7*^/#^	1.3 ± 0.2^$^	1.1 ± 0.2^$^	2.4 ± 0.3^$/^*	0.3 ± 0.1^$^
**IFN-γ** in the **alveolar septa**	0.7 ± 0.2	2.9 ± 0.3 *	2.5 ± 0.4*	3.2 ± 0.3*	0.5 ± 0.2^$^	1.2 ± 0.2^$^	2.9 ± 0.4*	0.7 ± 0.2^$^

The numbers of eosinophils in the alveolar septa are shown in Figure 5. There was an increase in the number of eosinophils in the OVA and ACO groups compared to those in animals exposed to saline solution. The number of eosinophils in the alveolar septa was lower in the treatment groups than in the ACO group (*p* < 0.05). In fact, there was a significant decrease in the number of eosinophils in the treatment groups.

### 3.5 Extracellular matrix remodeling

**FIGURE 6:**
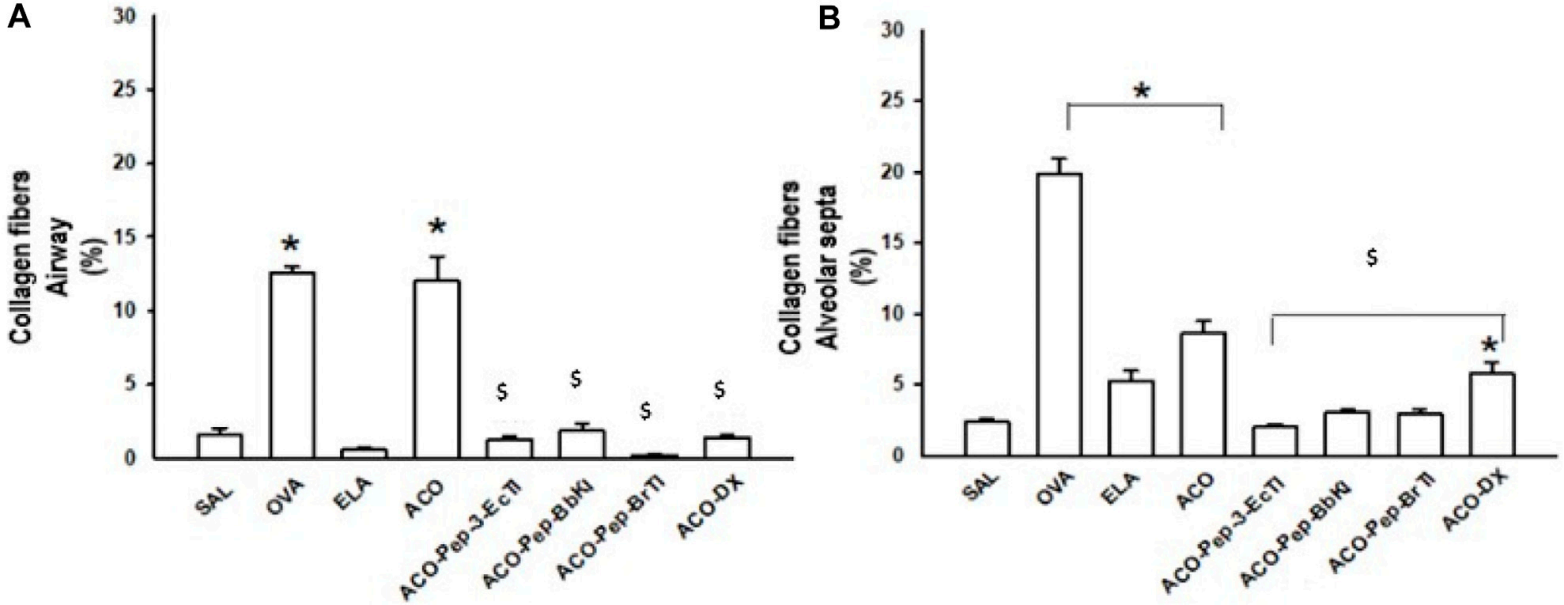
Effects of dexamethasone and peptide treatments on collagen fibers in the SAL, OVA, ELA, ACO, ACO-Pep-3-EcTI, ACO-Pep-BbKI, ACO-Pep-BrTI, and ACO-DX groups; ^*^ *p* < 0.05 compared to the SAL group; ^$^ *p* ≤ 0.05 compared to the ACO group.

Table 5 presents remodeling markers, such as MMP-9, MMP-12, and TGF-β, and Figure 6 illustrates collagen fibers.

**TABLE 5:** MMP, matrix metalloproteinase; TGF, transforming growth factor. The results are expressed in positive cells/10^4^µm^2^^*^ *p* < 0.05 compared to the SAL group; ^$^ *p* ≤ 0.05 compared to the ACO group; ^#^ *p* ≤ 0.05 for the ACO group compared to the OVA and ELA groups.

Extracellular matrix remodeling								
Remodeling (cells/10⁴µm^2^)	SAL	OVA	ELA	ACO	ACO-Pep-3-EcTI	ACO-Pep-BbKI	ACO-Pep-BrTI	ACO-DX
**TGF-β** in the **airway**	1.0 ± 0.3	5.6 ± 1.2^*^	2.8 ± 0.4	7.5 ± 1.0^*^	4.3 ± 0.6^*/$^	4.0 ± 0.4^*/$^	4.2 ± 0.5^*/$^	3.7 ± 0.4^*/$^
**TGF-β** in the **alveolar septa**	0.2 ± 0.1	4.8 ± 0.3^*^	3.0 ± 0.5^*^	10.0 ± 3.0^*/#^	2.0 ± 0.3^$^	2.4 ± 0.3^$^	2.6 ± 0.3^$^	1.0 ± 0.3^$^
**MMP-9** in the **airway**	0.2 ± 0.03	3.0 ± 0.1^*^	5.3 ± 0.2^*^	10.3 ± 0.3^*/#^	2.4 ± 0.2^*/$^	1.8 ± 0.1^*/$^	2.1 ± 0.1^*/$^	2.1 ± 0.1^*/$^
**MMP-9** in the **alveolar septa**	0.7 ± 0.1	10.5 ± 2.6^*^	6.3 ± 0.7^*^	8.7 ± 0.3^*/#^	2.6 ± 0.2^*/$^	1.9 ± 0.1^*/$^	1.8 ± 0.1^*/$^	2.4 ± 0.1^*/$^
**MMP-12** in the **airway**	1.8 ± 0.03	2.0 ± 0.3^*^	2.1 ± 0.4^*^	3.5 ± 0.3^*^	3.2 ± 0.4^*/$^	1.9 ± 0.2^*/$^	2.1 ± 0.3^*/$^	0.9 ± 0.2^*/$^
**MMP-12** in the **alveolar septa**	0.5 ± 0.1	0.2 ± 0.1	2.7 ± 0.4^*^	5.7 ± 0.5^*/#^	2.3 ± 0.3^*/$^	2.6 ± 0.3^*/$^	4.3 ± 0.6^*/$^	2.2 ± 0.3^*/$^

In the analysis of MMP-9 in both the airways and alveolar septa, there was an increase in these positive cells in the OVA, ELA, and ACO groups when compared to the SAL group (*p* < 0.05 for all comparisons). The ACO group showed a higher number of positive cells than the OVA and ELA groups. In contrast to the ACO and SAL groups, the ACO-Pep-3-EcTI, ACO-Pep-BbKI, ACO-Pep-BrTI, and ACO-DX groups showed attenuated changes (*p* < 0.05 for all comparisons).

When we look at the count of positive cells for MMP-12 both in the airways and in the alveolar septa, we observed an increase in the number of positive cells in the airways in the ACO group compared to the SAL group (*p* < 0.05 for all comparisons). The ACO group showed higher MMP-12 expression than the OVA and ELA groups. In the analysis of alveolar septa, there was an increase in these positive cells in the ELA and ACO groups compared to the SAL group (*p* < 0.05 for all comparisons). The ACO group showed a higher expression of MMP-12 positive cells than the OVA and ELA groups (*p* < 0.05 for both comparisons). When we analyzed the treatments, the ACO-Pep-BrTI and ACO-Pep-BbKI peptides and ACO-DX showed a total reversal of values, which was different from ACO (*p* < 0.05) and similar to that of SAL (*p* > 0.05). When we examined the alveolar septa in the ACO-Pep-3-EcTI, ACO-Pep-BrTI, ACO-Pep-BbKI, and ACO-DX treatment groups, we observed attenuation of the values, which differed from those of ACO and SAL.

We observed an increase in TGF-β cells in the ACO group when compared to the SAL group in both the airways and the alveolar septa. When we analyzed the treatments, we observed that in the airways, ACO-Pep-3-EcTI, ACO-Pep-BbKI, ACO-Pep-BrTI, and ACO-DX attenuated the alterations with a decrease in TGF-β compared to the ACO group (*p* < 0.05 for both comparisons) as these treatment groups were different from SAL. In the analysis of the alveolar septa, the ACO-PEP-EcTI, ACO-Pep-BbKI, and ACO-Pep-BrTI groups showed attenuated changes, in contrast to the ACO and SAL groups (*p* > 0.05 for all comparisons). The ACO-DX group completely reversed these changes, differing from ACO (*p* < 0.05) and being similar to SAL (*p* > 0.05).

In evaluating collagen fibers in the airways, we observed an increase in the OVA and ACO groups compared with the SAL group (*p* < 0.05 for both comparisons). The ACO-Pep-3-EcTI, ACO-Pep-BbKI, ACO-Pep-BrTI, and ACO-DX groups completely reversed these changes, differing from the ACO group (*p* < 0.05) and being similar to the SAL group (*p* > 0.05). In the evaluation of the alveolar septa, we observed an increase in the OVA, ELA, and ACO groups compared with the SAL group (*p* < 0.05 for both comparisons). The ACO-Pep-3-EcTI, ACO-Pep-BbKI, and ACO-Pep-BrTI groups completely reversed these changes, differing from the ACO group (*p* < 0.05) and being similar to the SAL group (*p* > 0.05). The ACO-DX group showed attenuated changes in collagen fibers, which were significantly different from those in the ACO (*p* < 0.05) and SAL groups (*p* > 0.05).

### 3.6 Oxidative stress response markers

**FIGURE 7:**
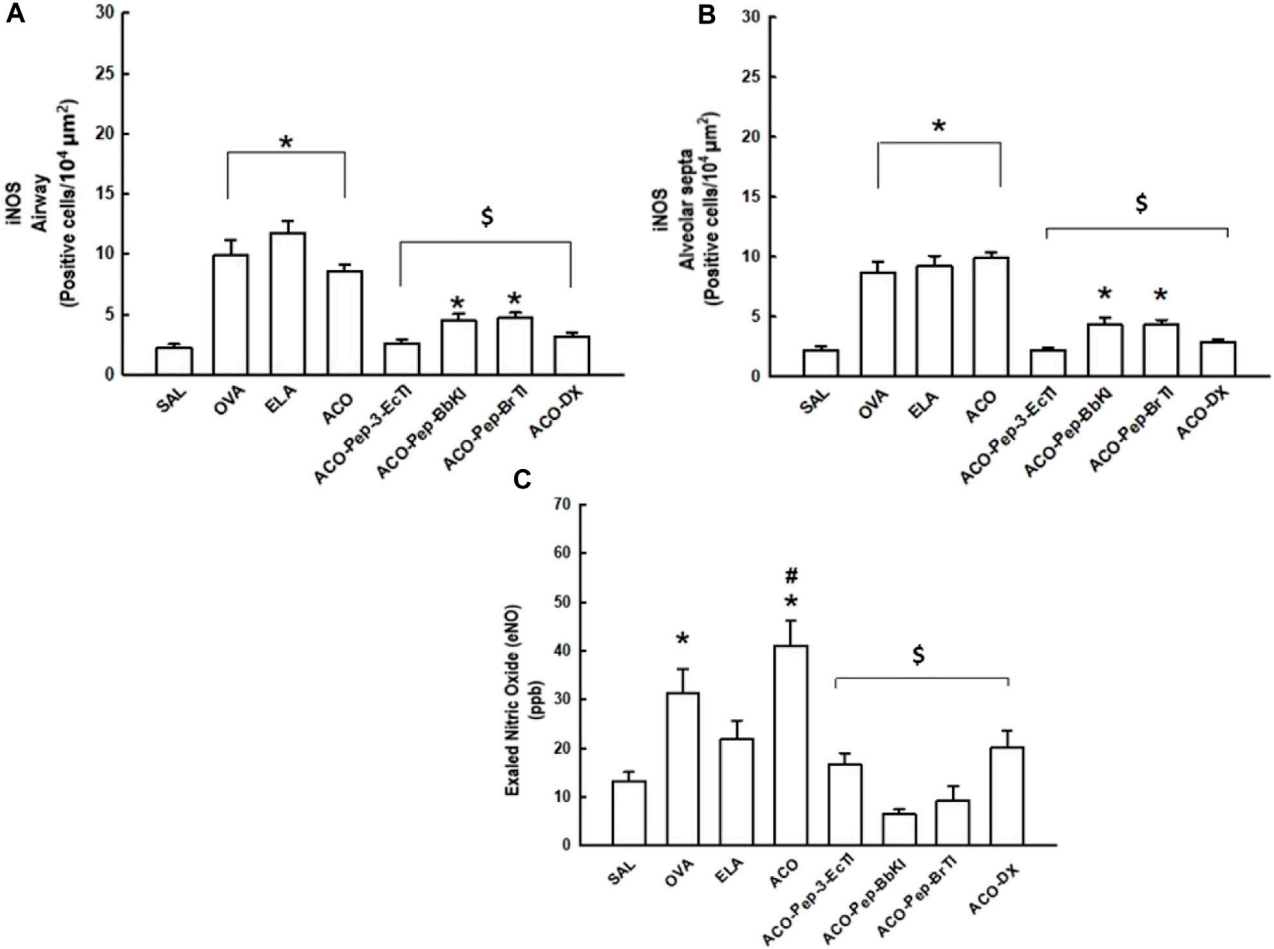
Effects of dexamethasone and peptide treatments on inducible nitric oxide synthase (iNOS) and exhaled nitric oxide (eNO) in the SAL, OVA, ELA, ACO, ACO-Pep-3pEcTI, ACO-Pep-BbKI, ACO-Pep-BrTI, and ACO-DX groups; *p < 0.05 compared to the SAL group; ^$^ *p* < 0.05 compared to the ACO group.

The results of oxidative stress markers are expressed in Figure 7. We observed an increase in iNOS in the ACO groups when compared to the SAL group (*p* < 0.05 for all comparisons). Both the airway and the alveolar septum were analyzed in the treatment groups. There was a total reversal of the alterations, where the results were different from those of the ACO (*p* < 0.05) group and similar to those of SAL (*p* > 0.05) in the ACO-Pep-3-EcTI and ACO-DX groups. The ACO-Pep-BbKI and ACO-Pep-BrTI groups showed attenuation of values, which was different from that of the ACO and SAL (*p* < 0.05 for all comparisons) groups.

#### 3.6.1 Exhaled nitric oxide

Figure 7 c) shows the assessment of exhaled nitric oxide (eNO). The OVA and ACO groups showed an increase in eNO compared with the SAL control group (*p* < 0.05 for both comparisons). In the ACO group, there was a potentiation of the increase in eNO when compared with the OVA and ELA groups (*p* < 0.05 for all comparisons).

The animals treated with DX and ACO-Pep-3-EcTI, ACO-Pep-BbKI, ACO-Pep-BrTI, and ACO-DX showed a reversal of eNO values as they were different from the ACO group and similar to the SAL group (*p* > 0.05).

The ACO-Pep-BbKI and ACO-Pep-BrTI groups showed lower values than the other treatment groups (ACO-Pep-3-EcTI and ACO-DX).

#### 3.6.2 Superoxide dismutase 3 (SOD3)

**FIGURE 8:**
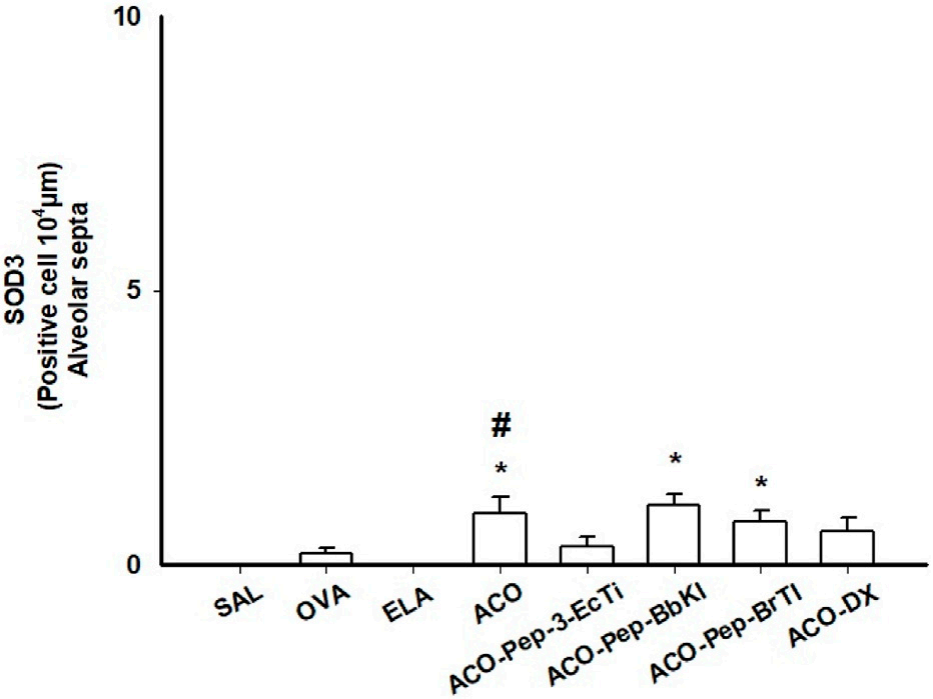
Comparison of SO3 levels in the SAL, OVA, ELA, ACO, ACO-Pep-3-EcTI, ACO-Pep-BbKI, ACO-Pep-BrTI, and ACO-DX groups; *p < 0.05 compared to the SAL group; ^#^ *p* < 0.05, ACO group compared to the OVA and ELA groups.

Analysis of Figure 8 revealed that the ACO group exhibited a significant increase in superoxide dismutase 3 (SOD3) levels compared to the SAL control group (*p* < 0.05), as well as a notable improvement compared to the OVA and ELA groups within the ACO group itself (*p* < 0.05 for all comparisons). Conversely, groups treated with ACO-Pep-3-EcTI, ACO-Pep-BbKI, ACO-Pep-BrTI, and ACO-DX showed no significant difference compared to the ACO group. However, both the ACO-Pep-BbKI and ACO-Pep-BrTI groups displayed significant differences compared to the SAL control group (*p* < 0.05 for all comparisons).

### 3.7 Signaling pathway

**FIGURE 9:**
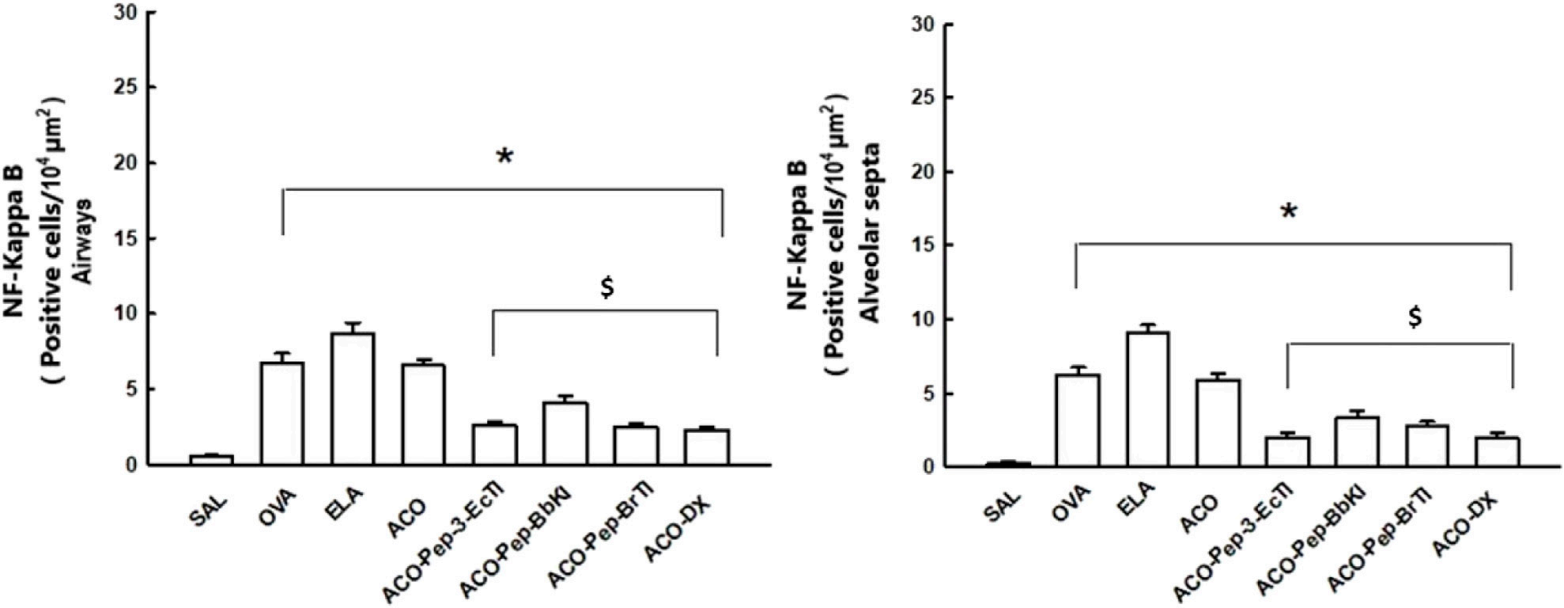
Graphs of the evaluation of signaling pathways in the SAL, OVA, ELA, ACO, ACO-Pep-3pEcTI, ACO-Pep-BbKI, ACO-Pep-BrTI, and ACO-DX groups; *p < 0.05 compared to the SAL group; ^$^ *p* < 0.05 compared to the ACO group; ^#^ *p* < 0.05, ACO group compared to the OVA and ELA groups.

Figure 9 shows the evaluation of the number of positive cells for NF-kappaB in the airways and alveolar septa of the SAL, OVA, ELA, ACO, ACO-Pep-3-EcTI, ACO-Pep-BbKI, ACO-Pep-BrTI, and ACO-DX groups. We observed an increase in these cells in the OVA, ELA, and ACO groups compared with the SAL group (*p* < 0.05 for all comparisons). In contrast to the ACO and SAL groups, the ACO-Pep-3-EcTI, ACO-Pep-BbKI, ACO-Pep-BrTI, and ACO-DX groups showed attenuated changes (*p* < 0.05 for all comparisons).

### 3.8 Qualitative analysis

**FIGURE 10:**
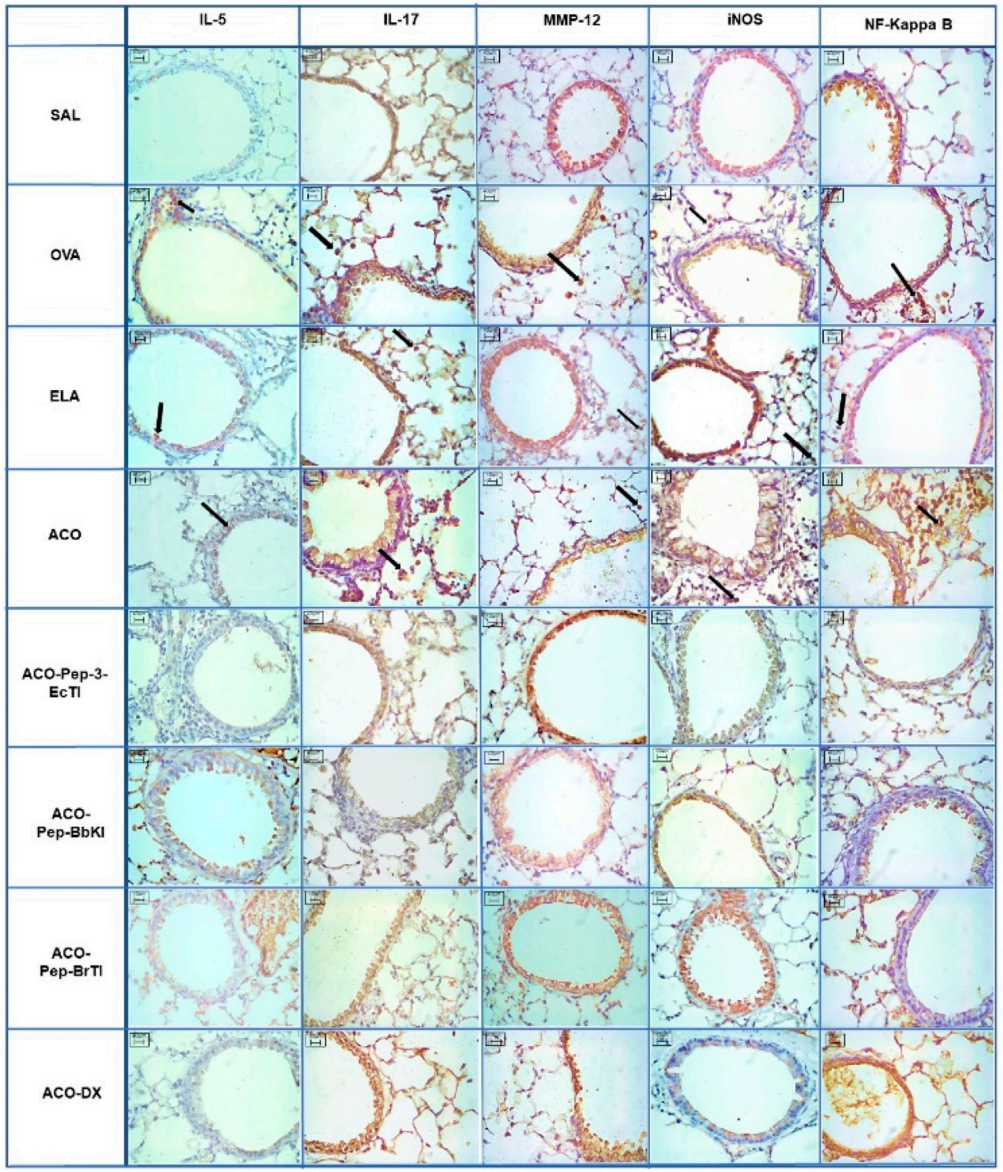
Qualitative airway analysis for inflammatory markers, remodeling markers, oxidative stress, and transcription factors. Microscopic photos of the results of immunohistochemical analyses showing the presence of inflammation around the airways. Magnification: 400×. The experimental groups are SAL, OVA, ELA, ACO, ACO-Pep-3-EcTI, ACO-Pep-BbKI, ACO-Pep-BrTI, and ACO-DX.

**FIGURE 11:**
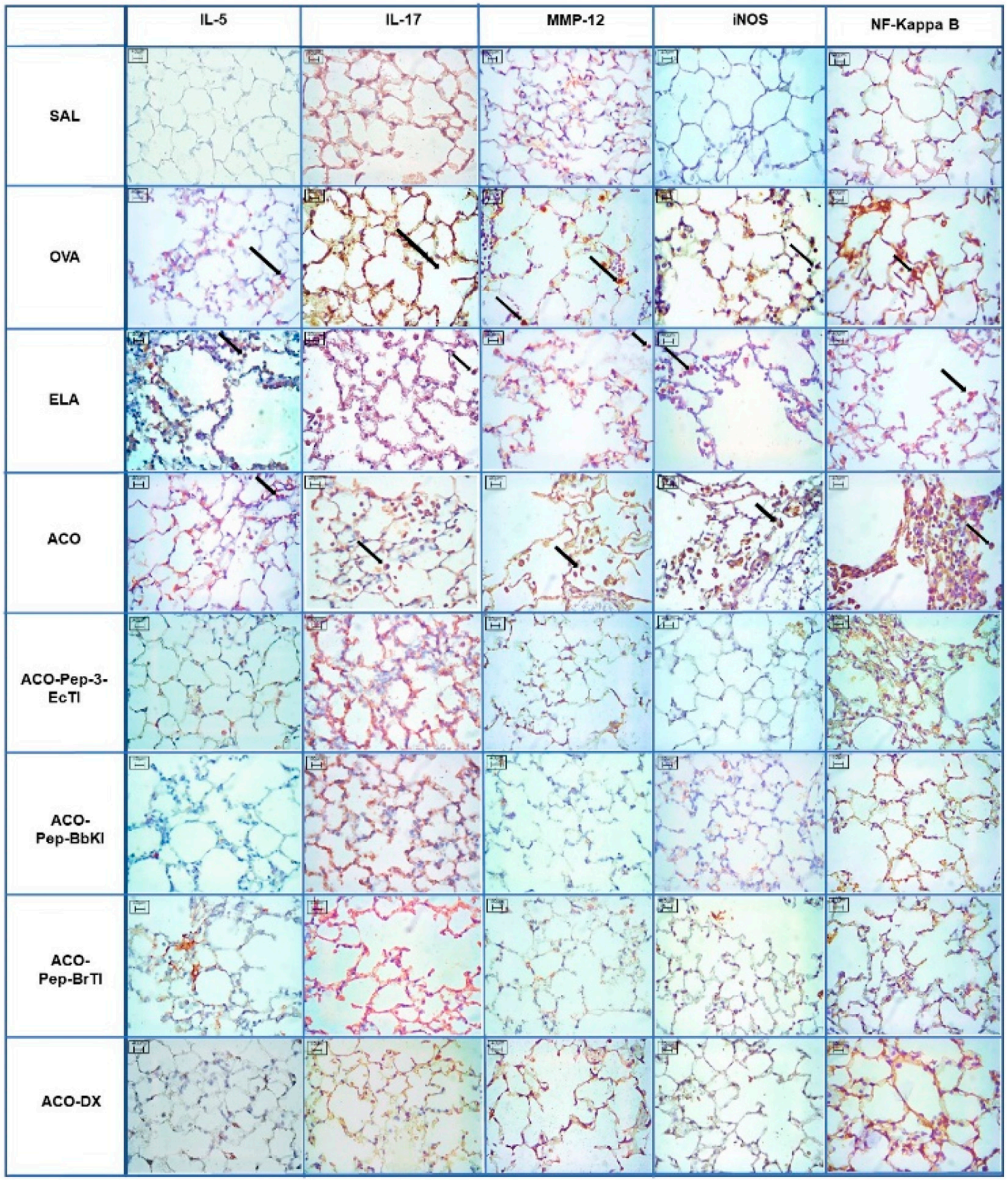
Qualitative alveolar septa analysis for inflammatory markers, remodeling markers, oxidative stress, and transcription factors. Microscopic photographs of the results of immunohistochemical analyses showing the presence of inflammation around the airways. Magnification: 400×. The experimental groups are SAL, OVA, ELA, ACO, ACO-Pep-3-EcTI, ACO-Pep-BbKI, ACO-Pep-BrTI, and ACO-DX.

Representative photomicrographs were taken to illustrate inflammatory processes, extracellular matrix remodeling, and oxidative stress in the airways; inflammatory processes are represented by IL-5 and IL-17, the characteristics of extracellular matrix remodeling by MMP-12, and markers of oxidative stress by iNOS and NF-kappaB. These photomicrographs are shown in Figure 10 and Figure 11, all of which were taken at 400× magnification.

## 4 Discussion

In this study, the effects of peptide (EcTI, BbKI, and BrTI) and DX treatments were evaluated using an experimental model of ACO. The sensitized animals, treated with plant protease inhibitors or DX, showed attenuation of pulmonary hyperresponsiveness and attenuation of the evaluated parameters related to the inflammatory response: control of alveolar septal injury, oxidative stress, reduction of remodeling extracellular matrix, and NF-kappaB, both in the airways and alveolar septa. In most analyses, we did not observe any differences between treatments.

Mouse studies are more commonly conducted using models of asthma or emphysema than an overlap of these two experimental models. Therefore, in the present study, the ACO model was adapted from the study by Ikeda et al. (2014), who used both intraperitoneal and inhaled OVA sensitization for the ACO model and PPE instillation, porcine pancreatic elastase intratracheally, for emphysema.

Ikeda et al. (2014) reported increased airway hyperresponsiveness and respiratory system compliance using the ACO model. Our study expanded this model by incorporating additional parameters, such as %Raw, %Htis, and %Gtis, in the methacholine dose–response curve, representing the viscoelastic properties of the respiratory system. We observed an exacerbation of the inflammatory response, increased alveolar spacing, and lung remodeling in the ACO model, making it a valuable tool for exploring the overlapping ACO model and potential therapeutic strategies.

The %Gtis analysis revealed a significant increase in the ACO group compared to that in both the OVA and ELA groups, indicating a heightened inflammatory response compared to that in the asthma model. A decrease in %Ers and %Htis compared to the OVA group was acceptable, given the ACO group’s characteristics related to the emphysema model (COPD). Additionally, the ACO group showed an increase in %Raw compared to the SAL group, justifying its intermediate characteristics in both the asthma and emphysema models.

Our study surpassed the parameters of Ikeda et al. (2014) by including the remodeling response and oxidative stress analyses, bolstering the ACO model. Notably, the ACO group exhibited a significant increase in eNO compared to the OVA and ELA groups, which was likely linked to elevated oxidative stress levels.

Among the main advantages of using an elastase model is the ability to rapidly induce emphysema with only one treatment using a low-cost reagent. This makes it a much more economical and easily administered option than 6 months of smoke exposure. Furthermore, disease severity can be controlled by adjusting the enzyme dose. In contrast to smoke-induced emphysema, severe emphysema caused by elastase instillation is relatively straightforward and facilitates the identification of lung function abnormalities. This is especially useful when assessing the effects of inhibitors or interventions such as lung volume reduction surgery or abnormalities in connective tissue and lung elasticity (Watanabe et al., 2004).

Our experimental groups, including those treated with plant protease inhibitors (ACO-Pep-3-EcTI, ACO-Pep-BbKI, and ACO-Pep-BrTI) and DX (ACO-DX), exhibited reduced %Rrs, %Raw, and %Gtis following the protocol. The observed decrease in hyperresponsiveness to methacholine indicated the bronchodilator action of both peptides and DX.

Our findings align with Rodrigues et al. (2019), who demonstrated a 21.5% reduction in %Rrs in mice sensitized with OVA treated with EcTI. When evaluating the elastance of the respiratory system (%Ers), the ACO group decreased compared to the OVA group and showed no significant difference compared to the ELA group. This finding supports the %Htis data, indicating impaired elastic recoil due to alveolar wall destruction and enlargement. Previous studies administering intratracheal elastase, such as ours, have reported increased Ers and Htis, which were reduced after treatment with serine protease inhibitors. However, our results revealed a reduction in the %Ers and %Htis in the ELA and ACO groups.

We hypothesized that the shorter time interval (7 days) between elastase instillation and pulmonary mechanics collection in our study may not have provided sufficient time for alveolar septa remodeling, leading to decreased %Ers and %Htis in the emphysema and overlap model (ACO) groups. Oliveira et al. (2016) emphasized the role of collagen and elastic fibers in enhancing lung tissue elastance and resistance proportional to their volume fraction increase in the ELA group. In our study, the treatment groups (ACO-DX, ACO-Pep-3-EcTI, and ACO-Pep-BbKI) completely reversed these alterations in the ACO group, resembling those in the control SAL group. This suggests attenuation of lung parenchymal destruction and improvement in elastic recoil. ACO-Pep-BrTI treatment did not differ significantly from ACO treatment but showed no difference from the SAL group.

Alveolar damage, a characteristic of emphysema, can be observed by an increased value of Lm as it comprises the measurement of the average space between the opposing alveolar walls (Global Initiative for Chronic Obstructive Lung Disease, 2022). In our study, we observed that the ACO group had higher values than the OVA and ELA groups, thus showing hyperdistension and destruction of alveolar septa associated with the remodeling of extracellular matrix components (Biselli et al., 2019). Studies with protease inhibitors, such as that by Lourenço et al. (2014), showed a decrease in Lm measurement after treatment, which corroborates our study, where the peptides and DX completely reversed the changes when compared to the ACO group, suggesting attenuation of lung injury.

Bronchoalveolar lavage analysis is considered one of the most effective methods for evaluating pulmonary inflammation (Churg and Wright, 2005). In our study, the treatment groups exhibited a significant decrease in the number of bronchoalveolar lavage cells, including neutrophils, macrophages, and eosinophils. Specifically, lymphocyte analysis revealed a significant decrease only in the ACO-Pep-3-EcTI and ACO-DX groups compared to the ACO group, indicating a notable anti-inflammatory effect of both plant protease inhibitors and DX. However, there were no significant differences between peptide and DX treatments.

There was a reduction in eosinophil concentration in the ACO-Pep-3-BbKI, ACO-Pep-BbKI, ACO-Pep-BrTI, and ACO-DX treatment groups, whereas treatment with BbKI and BrTI peptides did not attenuate the lymphocyte response. This aligns with the findings of Florencio et al. (2019) and Lourenço et al. (2014), who evaluated the effects of rBmTI-A (a serine protease inhibitor) in asthma and pulmonary emphysema models and observed a reduction in eosinophil concentration without a decrease in lymphocytes.

Cytokines play a crucial role in regulating and activating asthma, and as ACO shares some characteristics with asthma, this study demonstrates that the ACO-Pep-3-EcTI, ACO-Pep-BbKI, ACO-Pep-BrTI, and ACO-DX treatment groups exhibited a positive response in controlling inflammation. In the ACO group, the analysis of various cytokines, including IL-1β, IL-5, IL-6, IL-10 (airway), IL-13 (alveolar septa), INF-γ (airway), and TNF-α (alveolar septa), revealed increased values compared to ELA and OVA. IL-17 showed higher levels only when compared to the OVA group. These findings support those of Ikeda et al. (2014), who observed increased airway hyperresponsiveness, leukotriene levels, and CD4^+^ and CD8^+^ T cells in BALF in the ACO group, indicating greater pulmonary inflammation.

In the IL-1β analysis in the airways, both peptide and DX treatment groups attenuated the ACO response. While DX and the EcTI and BbKI peptides completely reversed the alveolar septa, the BrTI peptide did not show a significant reduction. EcTI and BbKI peptides were as effective as DX.

In our study, treatment with both the peptides and DX attenuated the concentration of IL-5 compared to that in the ACO group. In the airway analysis, BbKI and BrTI peptides completely reversed inflammation, and in the analysis of the alveolar septa, all peptides showed reversion, similar to the SAL group. This indicated a reduction in eosinophilic inflammation, which prevented potential exacerbations. Notably, the peptide groups differed from the ACO-DX group only in the airway analysis but were smaller, demonstrating their effectiveness.

Analysis of IL-4 in the airways revealed that the treatment groups were not attenuated relative to the ACO group, whereas attenuation of the inflammatory response in the alveolar septa to ACO was observed only in the ACO-Pep-3-EcTI and ACO-DX groups.

IL-10 plays a crucial role in modulating the immune system by exerting compensatory and anti-inflammatory effects. IL-10 is constitutively produced in healthy airways, preventing the expression of B7 in alveolar macrophages and protecting against inflammatory responses to allergens. In addition, it regulates allergic diseases by inhibiting eosinophil and IgE synthesis, demonstrating its ability to neutralize airway inflammation in experimental models. IL-10 inhibits cytokines associated with cellular immunity and allergic inflammation while stimulating humoral and cytotoxic immune responses. In pathological conditions, such as asthma, IL-10 production decreases, indicating a possible relationship between its deficiency and asthmatic inflammation. IL-10 acts as a cytokine inhibitor, influencing both the Th1 and Th2 states and modulating the IL-1 pathway, suggesting a negative compensatory mechanism in the inflammatory response. IL-10 is relevant in both asthma (associated with eosinophils) and COPD (associated with neutrophils), indicating a possible impaired resolution of inflammation in these conditions. A previous study suggested a significant decrease in the number of IL-10-positive cells in patients with COPD and asthma. In the ACO model, IL-10 showed an unexpected increase, possibly indicating a compensatory mechanism in the face of intensified inflammation. Peptides appear to modulate this increase, highlighting their therapeutic potential for managing inflammation in the ACO model (Rosenwasser and Borish, 1997; Denys et al., 2002; Ogawa et al., 2008).

Our findings align with those of Rodrigues et al. (2019), who demonstrated a reduction in IL-5, IL-6, and IL-13 levels. Similarly, Florencio et al. (2019) observed comparable attenuation of IL-5, IL-10, IL-13, and IL-17 levels using the protease inhibitor rBmTI-A.

In our analysis, the cytokines IL-6, IL-10, IL-13, and IL-17 in the groups treated with peptides showed similar results to those of the treatment with corticosteroids, reducing the values compared with the ACO group, which was not different from the SAL group. This attests to the effectiveness of these treatments, alluding to promising results for the use of these peptides as possible drugs.

We showed that, in relation to oxidative stress, there was a reduction in eNO and iNOS (airways and alveolar septa) in the ACO-Pep-3-EcTI, ACO-Pep-BbKI, ACO-Pep-BrTI, and ACO- DX groups when compared to the ACO group, with no difference between treatment groups. Regarding the lung remodeling response, in the analysis of MMP-9, MMP-12, and TGF-β, we observed attenuation of the treated groups in relation to the ACO group in both the airways and the alveolar septa.

In agreement with our findings, Theodoro-Junior et al. (2017) demonstrated in an emphysema model that an EcTI inhibitor mitigated oxidative stress, leading to decreased cellular expression of eNO and iNOS in both the alveolar septa and airway pathways. Similarly, Rodrigues et al. (2019) reported a 66% reduction in iNOS-positive cells in airways and a 56% reduction in alveolar septa following EcTI treatment in ovalbumin-sensitized animals, attributed to decreased eosinophilic inflammation and inflammatory cytokine expression. Martins-Olivera et al. (2016) observed a decreased response in the BbKI-treated group, marked by reduced expression of iNOS and eNO-positive cells in both the alveolar and airway septa.

MMP-12 has been studied and described as a metalloprotease released mainly by macrophages. It may be an important elastolytic enzyme responsible for emphysematous lesions in rodents. Our study corroborates the findings of Florencio et al. (2019), who demonstrated that treatment with peptides (EcTI, BbKI, and BrTI) and DX was sufficient to reduce the number of MMP-12 positive cells, which could explain the inhibitory effect of DX and peptides on the destruction of the lung parenchyma.

Oxidative stress plays a significant role in the pathogenesis of asthma and COPD, mediated in part by the activity of superoxide dismutases (SODs), pivotal antioxidant enzymes that neutralize reactive oxygen species (ROS), such as superoxide, thereby safeguarding cells against oxidative damage (Poonyagariyagorn et al., 2014; Lewis et al., 2021). Our study elucidates that in patients with ACO, levels of SOD3 are heightened compared to the SAL control group, indicative of an adaptive response to mitigate the oxidative stress associated with ACO. Notably, SOD3 assumes a critical role in airway antioxidant defense, with its elevation suggestive of a concerted effort to mitigate cellular damage and inflammation resulting from oxidative stress. The ramifications of the oxidative stress response may hinge upon an imbalance between elevated oxidants, diminished antioxidants, or a combination thereof, which could significantly influence the progression of respiratory ailments. Contrary to expectations, our findings did not unveil significant antioxidant effects amidst the observed oxidative stress. This may imply either an organismal incapacity to effectively counteract heightened oxidants or an inadequate antioxidant response to oxidative insult.

NF-kappaB plays a crucial role in oxidative stress, as excessive activation perpetuates the production of inflammatory mediators in severe asthma and may exacerbate COPD, given its association with TNF-α, IL-8, and other inflammatory cells. Notably, the expression of iNOS and cytokines related to the Th2 and Th17 profiles relies on NF-kappaB transcription (Fukusaki, 2019). In our study, the experimental groups (ACO, ELA, and OVA) exhibited elevated NF-kappaB values, and the treatments were effective in reducing these values in the ACO group. Previous studies utilizing plant protease inhibitors in experimental models of asthma and emphysema did not include an analysis of NF-kappaB values (Oliva et al., 2015; Almeida-Reis et al., 2017; Theodoro-Júnior et al., 2017; Rodrigues et al., 2019).

This study has limitations as a toxicity profile analysis of the inhibitors used was not conducted. However, previous studies employing inhibitors such as EcTI, BbKI, BbCI, and rBmTI-A in other experimental models have not revealed any adverse effects (Oliva et al., 2015; Almeida-Reis et al., 2017; Theodoro-Júnior et al., 2017; Florencio et al., 2019). Despite the importance of treatments with Pep-3-EcTI, Pep-BbKI, and Pep-BrTI in influencing lung mechanics, pulmonary inflammatory response, extracellular matrix remodeling, and oxidative stress, it is crucial to note that as natural compounds of plant origin with protease inhibitory activity, caution is warranted when extrapolating these findings to humans. As a limitation, we noted the absence of systemic lung analysis. We chose to perform immunohistochemical analyses in different lung compartments, such as the airways and alveolar septa, because of their relevance to the specific research objectives. Additional studies are essential for safety assessment, understanding the mechanisms of action, and considering their clinical applications.

## 5 Conclusion

In the experimental model of ACO used in this study, we concluded that the peptides under investigation (PEP-3-EcTI, Pep-BbKI, and Pep-BrTI) yielded similar results to DX, which is currently the gold standard treatment for ACO patients in terms of oxidative stress, inflammatory markers, lung function, and extracellular matrix remodeling. Thus, the inhibitors attenuated bronchial hyperresponsiveness, inflammatory response, extracellular matrix remodeling, and oxidative stress induced by both OVA and intratracheal elastase in the lungs of this experimental model.

These findings are cutting-edge, and as such, we can conclude that plant protease inhibitors may be a promising therapeutic strategy for ACO treatment, although further studies are required to elucidate the mechanisms involved.

## Data Availability

The original contributions presented in the study are included in the article/Supplementary Material; further inquiries can be directed to the corresponding author.

## Abbreviations

BALF, bronchoalveolar lavage fluid; BSA, bovine serum albumin; DX, dexamethasone; ELA, elastase; H&E, hematoxylin and eosin; Ig, immunoglobulin; IgE, immunoglobulin E; IHC, immunohistochemistry; IL, interleukin; iNOS, inducible nitric oxide synthase; MMPs, matrix metalloproteinases; NF-κB, nuclear factor kappa-light-chain-enhancer of activated B cells; OVA, ovalbumin; PBS, phosphate-buffered saline; Pep-3-EcTI, peptide derived from *E. contortisiliquum* trypsin inhibitor; Pep-BbKI, peptide derived from kallikrein inhibitor isolated from *B. bauhinioides*; Pep-BrTI, *B. rufa* peptide modified from *B. bauhinioides*; PPE, porcine pancreatic elastase; Th2, T helper type 2; TGF-β, transforming growth fator; TNF-α, tumor necrosis factor alpha.
